# Protein-targeted corona phase molecular recognition

**DOI:** 10.1038/ncomms10241

**Published:** 2016-01-08

**Authors:** Gili Bisker, Juyao Dong, Hoyoung D. Park, Nicole M. Iverson, Jiyoung Ahn, Justin T. Nelson, Markita P. Landry, Sebastian Kruss, Michael S. Strano

**Affiliations:** 1 Department of Chemical Engineering, Massachusetts Institute of Technology, 77 Massachusetts Avenue, Cambridge, Massachusetts 02139, USA; 2 Department of Biological Systems Engineering, University of Nebraska-Lincoln, 223 L.W. Chase Hall, Lincoln, Nebraska 68583, USA; 3 Department of Chemical and Biomolecular Engineering, University of California Berkeley, 201 Gilman Hall, Berkeley, California 94720, USA; 4 Department of Chemistry, Institute for Physical Chemistry, Göttingen University, Tammannstrasse 6, 37077 Göttingen, Germany

## Abstract

Corona phase molecular recognition (CoPhMoRe) uses a heteropolymer adsorbed onto and templated by a nanoparticle surface to recognize a specific target analyte. This method has not yet been extended to macromolecular analytes, including proteins. Herein we develop a variant of a CoPhMoRe screening procedure of single-walled carbon nanotubes (SWCNT) and use it against a panel of human blood proteins, revealing a specific corona phase that recognizes fibrinogen with high selectivity. In response to fibrinogen binding, SWCNT fluorescence decreases by >80% at saturation. Sequential binding of the three fibrinogen nodules is suggested by selective fluorescence quenching by isolated sub-domains and validated by the quenching kinetics. The fibrinogen recognition also occurs in serum environment, at the clinically relevant fibrinogen concentrations in the human blood. These results open new avenues for synthetic, non-biological antibody analogues that recognize biological macromolecules, and hold great promise for medical and clinical applications.

## Abstract (web summary)

Corona phase molecular recognition (CoPhMoRe) involves the deposition of a heteropolymer onto a nanoparticle surface, providing a recognition site for a given analyte. Here, the authors show that CoPhMoRe can be used to selectively detect proteins (fibrinogen) with high selectivity, including in a complex serum environment.

## Graphical abstract

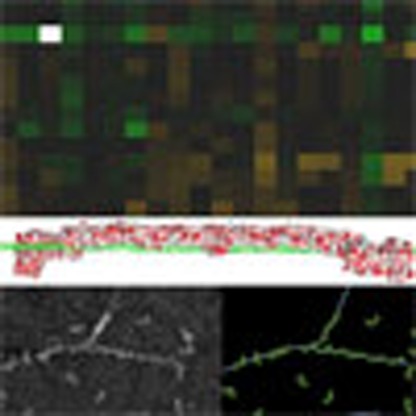

Molecular recognition elements are central to a wide variety of applications, including chemical assays and sensors^1^,^2^, catalysis^3^ and directed assembly of nanoparticles^4^,^5^,^6^. The most advanced, generic molecular recognition schemes involve natural systems, including antibodies^7^ and aptamers^8^. Molecular imprinting^3^ is one example of a purely synthetic recognition scheme, however, imprinting of biological macromolecules, such as proteins, remains challenging^9^,^10^. We have recently introduced a concept that we term corona phase molecular recognition (CoPhMoRe) as a generic molecular recognition scheme using a nanoparticle surface to template a heteropolymer. An adsorbed phase of a surfactant or a polymer on a nanoparticle, called the corona, and normally selected from a library of such molecules, is necessarily constrained and structured by the molecular interactions with the nanoparticle surface. CoPhMoRe is achieved when a heteropolymer–nanoparticle hybrid selectively binds a target analyte owing to the structure adopted by the polymer when folded onto the particle surface. In practice, a CoPhMoRe screen of a heteropolymer or surfactant library is accelerated if the underlying nanoparticle has an optical response to the molecular binding event, allowing for high-throughput detection of selective phases. Our work to date has used near-infrared (nIR) fluorescent single-walled carbon nanotubes (SWCNT) as an underlying reporter of this molecular interaction^11^,^12^,^13^,^14^, where non-covalent functionalization^12^,^13^,^14^,^15^,^16^,^17^,^18^,^19^,^20^ was used to produce distinct corona phases.

CoPhMoRe screening^16^,^17^ for small molecules normally proceeds with the construction of a heteropolymer library such that each element can suspend the nanoparticle (SWCNT in this case) creating an array of colloidal dispersions. The specific synthetic polymers for library screening necessarily have hydrophobic segments or moieties that adsorb onto the hydrophobic surface of the SWCNT, pushing hydrophilic segments into solution. The composition of the polymer controls the specific configuration, either static or dynamic, that can recognize a target analyte of interest. For small molecules, we have previously shown that the interaction between the polymer, nanotube and analyte can be described using a two-dimensional (2D) equation of state model, allowing reasonably accurate prediction of molecular recognition^21^. In the case of SWCNT, high-throughput fluorescence spectroscopy that scans for spectral changes associated with analyte binding as either fluorescent intensity or emission wavelength modulation can then be used to identify CoPhMoRe phases. Previous work has demonstrated CoPhMoRe SWCNT sensors for riboflavin, L-thyroxine, and estradiol, using boronic acid-substituted phenoxy dextran, polyethylene glycol (PEG) brush, and rhodamine isothiocyanate difunctionalized-PEG SWCNT coronae, respectively^16^. In addition, a variety of DNA oligonucleotides demonstrated discrimination among a panel of neurotransmitters^17^. Despite initial success with small organic molecules detection, CoPhMoRe has not yet been adapted or demonstrated for macromolecules, with an open question of whether such coronae are capable of the large area, selective interactions necessary to discriminate between soluble proteins.

Although antibodies can be raised to identify both small and macromolecular targets alike, the need of a living organism for production poses a limitation in high-throughput exploratory research^22^. In principle, a pinned configuration of a specific polymer could be found such that it maps the contours and localized chemical affinities of a certain face of a protein analyte^23^, but experimental realization of this has not yet been demonstrated. As a synthetic approach for recognizing biological molecules, it is possible that protein CoPhMoRe would find extensive use in a variety of sensor and assay arrangements where degradation, stability, cost and production scale prevent natural recognition elements from being employed.

In this work, we adapt the CoPhMoRe screen to macromolecules, developing a series of validation assays to unequivocally assign fluorescence modulation to CoPhMoRe phase binding and recognition. We construct the first library and conduct a screening of CoPhMoRe phases against 14 protein analytes, selected for their abundance and clinical significance in human whole blood^24^,^25^,^26^. The human plasma proteome consists of hundreds of proteins whose concentrations span over >10 orders of magnitude^27^, from which we selected albumin, immunoglobulin G (IgG), fibrinogen, α1-antitrypsin, transferrin, haptoglobin, α2-macroglobulin, immunoglobulin A (IgA), immunoglobulin M (IgM), α1-acid-glycoprotein and apolipoprotein A-I, owing to their high abundancy, and insulin, human chorionic gonadotropin and C-reactive protein, owing to their clinical importance. Despite our limited initial library size, a screen of 20 distinct SWCNT corona phases against the protein panel shows surprising recognition of fibrinogen to the exclusion of the other 13 using a dipalmitoyl-phosphatidylethanolamine (DPPE)-PEG (5 kDa)—SWCNT CoPhMoRe phase. We show that this recognition is not related to isoelectric point, aggregation, molecular weight, protein hydrophobicity or any other non-selective mechanism. Further, we demonstrate that the fibrinogen recognition still occurs in competitive binding assays, starting from testing in the presence of albumin, which is a common agent for surface passivation and blocking of non-specific binding^28^,^29^,^30^, followed by a successful demonstration of fibrinogen detection in serum environment. We hypothesize that a combination of the SWCNT corona phase formed by the specific phospholipid-PEG along with the unique three-dimensional conformation of the fibrinogen protein, a high aspect ratio elongated molecule, is responsible for the molecular recognition. This is supported by high-resolution atomic force microscopy (AFM) images, which manifest the facile recognition of fibrinogen on the CoPhMoRe phase by physical binding and the alignment of the fibrinogen molecules with the nanotube axis, and by quartz crystal microbalance with dissipation (QCM-D) measurements, which show fibrinogen monolayer adsorption on top of the SWCNT layer such that the protein molecules lay with their long axis parallel to the nanotube surface layer. In addition, kinetic measurements confirm a three-step molecular adsorption, consistent with the three nodule structure of the fibrinogen^31^,^32^. Moreover, photo-absorption measurements and cryo-transmission electron microscope (TEM) imaging rule out aggregation or precipitation of the SWCNT, which is supported by a demonstration of the sensor functionality while encapsulated within a thin hydrogel bed or when adsorbed onto a surface. The functionality at the single sensor level of individual surface-immobilized SWCNT, along with the optical read-out in real-time, enables a detection with both spatial and temporal resolution. These results open the door to protein CoPhMoRe-based recognition for proteomic and medical research, while expanding the applicability of the CoPhMoRe concept to bio-macromolecules for the first time.

## Results

### SWCNT suspension characterization

**Figure 1:**
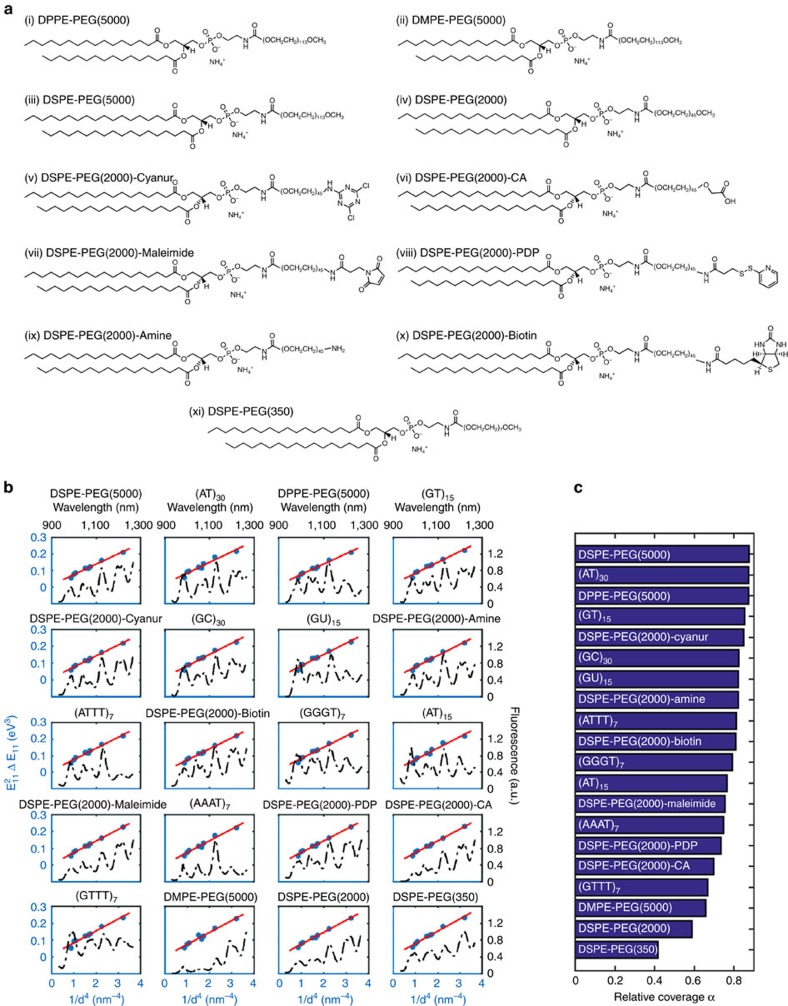
SWCNT suspension library. (**a**) The structure of the phospholipid-PEG constructs used in this study to suspend the SWCNT. (i) DPPE-PEG(5000), (ii) DMPE-PEG(5000), (iii) DSPE-PEG(5000), (iv) DSPE-PEG(2000), (v) DSPE-PEG(2000)-Cyanur, (vi) DSPE-PEG(2000)-carboxylic acid (CA), (vii) DSPE-PEG(2000)-Maleimide, (viii) DSPE-PEG(2000)-[3-(2-Pyridyldithio)-propionyl] (PDP), (ix) DSPE-PEG(2000)-Amine, (x) DSPE-PEG(2000)-Biotin, (xi) DSPE-PEG(350). The number in parentheses is the molecular weight of the PEG chain in Daltons. (**b**) The solvatochromic shift as a function of the SWCNT diameter to the power of negative 4 (*d*^−4^, blue dots) and its linear fit (solid red curve), and the fluorescent emission intensity for each wrapping (dashed black curve). (**c**) The relative surface coverage of the polymer wrappings ranked in descending order.

SWCNT produced by the HiPCO catalysis, were suspended with ssDNA and ssRNA by direct sonication^33^, or with phospholipid-PEG polymers (listed in Fig. 1a), by exchanging sodium cholate (SC) wrapping using dialysis. Successful suspensions were evident from the distinct absorption peaks (Supplementary Fig. 1a) and the bright fluorescent emission under 785 nm laser excitation (dashed black curve in Fig. 1b and Supplementary Fig. 1b). In addition, when the excess phospholipid-PEG molecules were removed from the solution by filtration or dialysis, the suspension retained its stability, manifested by the absorption and fluorescent spectra, as opposed to the initial SC-SWCNT suspension, which underwent massive aggregation and lost both its absorption and fluorescence emission (Supplementary Fig. 2).

The fluorescent emission^34^ peaks correspond to the radiative decay of excitons^35^, which are influenced by the local dielectric environment of the SWCNT surface^36^. Fluctuating electric fields resulting from random orientations of dipole moments in the close proximity of the SWCNT can induce a dipole moment on the highly polarizable SWCNT. Although SWCNT have no net dipole moment, the electric fields can cause a shift in their electronic transitions (solvent Stark effect). The semi-empirical functional form describing this shift is given by^36^:


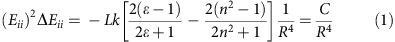


where *E*_*ii*_ is the optical transition energy, Δ*E*_*ii*_ is the difference between the optical transition energy in the dielectric environment and the optical transition energy of pristine SWCNT in air, *L* is a fluctuation factor, *k* is a scaling constant of the SWCNT polarizability, *ɛ* is the static dielectric constant, *n* is the refractive index and *R* is the nanotube radius. The constant *C* gathers all the parameters that are constant for a specific chirality.

The experimental optical transitions of the various SWCNT suspensions, *E*_11_, were calculated by deconvoluting the fluorescent emission spectra to the different chiralities in the HiPCO sample^37^ (Supplementary Fig. 3), and the optical transitions in air were calculated according to^36^:


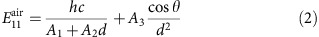


where *h* is Planck's constant, *c* is the speed of light, *d* is the SWCNT diameter, *θ* is the chiral angle corresponding to the SWCNT chirality (*n*, *m*), *A*_1_=61.1 nm and *A*_2_=1,113.6. By noting mod((*n*−*m*), 3)=*j*, *A*_3_=−0.077 eV nm^2^ for *j*=1 and *A*_3_=0.032 eV nm^2^ for *j*=2.

The solvatochromic shift, (*E*_11_)^2^Δ*E*_11_, is plotted against the SWCNT diameter to the power of negative 4 (*d*^−4^) for the various chiralities in each suspension in Fig. 1b, and the data is fitted with a linear curve (blue dots, and solid red curve, respectively). The slopes of the fit curves and the corresponding R-squared parameters are listed in Supplementary Table 1. These slopes can be used to estimate the effective dielectric constant, *ɛ*_eff_, in the vicinity of the SWCNT, by comparing them to the slope of a reference system^38^,^39^ of SWCNT suspended in *N*-methyl-2-pyrrolidone (NMP)^36^:


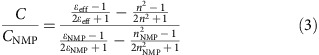


where *C*_NMP_=0.060 eV^3^ nm^4^, *ɛ*_NMP_=32.2 and *n*_NMP_=1.47 (Supplementary Table 1). We assume that the refractive indexes of the DNA, RNA and phospholipid-PEG wrappings are equal to that of water (*n*=1.333). On the basis of the effective dielectric constant, we can estimate the relative surface coverage of the SWCNT, *α*, by its wrapping, by assuming a linear combination of the surrounding water (*ɛ*_water_=88.1) and the wrapping polymer (*ɛ*_p_) contributions:


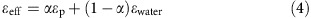


where *ɛ*_p_=4 for DNA and RNA wrappings^40^ and *ɛ*_p_=2.08 for the phospholipid wrappings^39^. The parameter *α* was used to rank the polymer wrappings in descending order (Fig. 1c). As we show below, the molecular recognition of fibrinogen occurs for DPPE-PEG(5000) to the exclusion of the others, and this CoPhMoRe phase has one of the highest polymer surface coverages.

### Protein library screening

**Figure 2:**
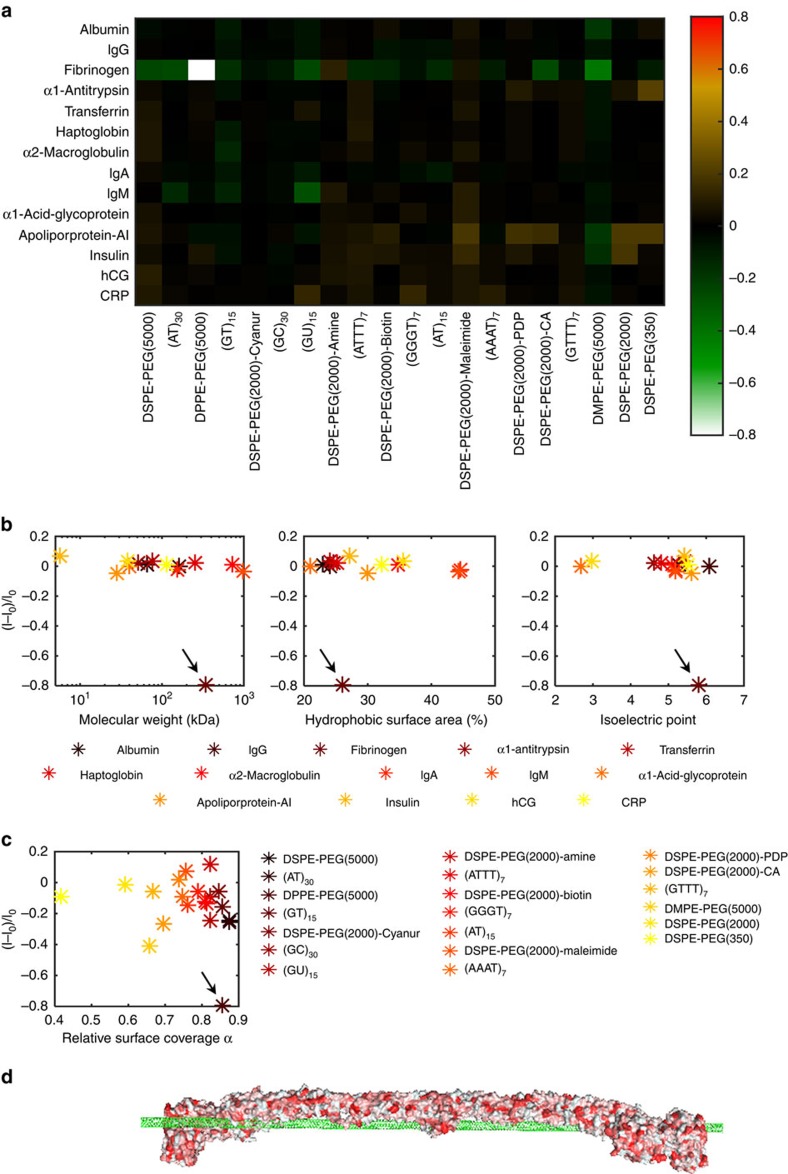
Protein CoPhMoRe screen. (**a**) Heat-map of the normalized response of the joint peak of the (9,4) and (7,6) SWCNT chiralities to the various proteins. The SWCNT and proteins concentrations were 1 mg l^−1^ and 20 μg ml^−1^, respectively. (**b**) The normalized emission intensity response (*I*−*I*_0_)/*I*_0_, where *I* and *I*_0_ are the final and initial fluorescent intensities, respectively, of DPPE-PEG(5000)-SWCNT versus the protein parameters: molecular weight, relative hydrophobic surface area and isoelectric point. The black arrows point to the fibrinogen data points. (**c**) The normalized emission intensity response of the various SWCNT suspensions to fibrinogen versus the relative surface coverage. The black arrow points to the DPPE-PEG(5000) data point. Each value represents the average of three replicates. (**d**) Illustration of the possible fibrinogen—SWCNT interaction. The fibrinogen surface is coloured according to the hydrophobicity of each amino acid ranging from white (hydrophilic) to red (hydrophobic).

We have constructed a library of 14 proteins of human origin, some of which are highly abundant in blood^27^ (such as albumin, IgG, fibrinogen, α1-antitrypsin, transferrin, haptoglobin, α2-macroglobulin, IgA, IgM, α1-acid-glycoprotein and apolipoprotein A-I), whereas the others are relatively rare but of clinical significance (insulin^41^, human chorionic gonadotropin^42^ and C-reactive protein^43^). The responses of the fluorescent emission of the DNA, RNA and phospholipid-PEG SWCNT suspensions were recorded following an hour incubation with each of the proteins at 20 μg ml^−1^ in phosphate buffered saline (PBS). The normalized fluorescence response of the joint peak of the (9,4) and (7,6) tubes, which is readily distinguishable, (Supplementary Fig. 1b) is presented in a heat-map in Fig. 2a.

Manifested in the heat-map, the DPPE-PEG(5000)-SWCNT (Fig. 1a (i)) shows an obvious distinguishable response to fibrinogen, of an 80% decrease in fluorescence intensity, whereas other proteins induce a negligible response of <5% for this complex. Hence, the DPPE-PEG(5000)-wrapped SWCNT can be considered as a selective sensor for fibrinogen.

Protein parameters such as molecular weight, relative hydrophobic surface area or isoelectric point, cannot explain the pronounced response of the DPPE-PEG(5000)-SWCNT fluorescence to fibrinogen, which appears as an outlier in plots of the normalized response as a function of these three variables (Fig. 2b).

The DPPE-PEG(5000) wrapping that renders the SWCNT a selective sensor for fibrinogen has the second highest relative coverage of the SWCNT surface (85.5%) among the phospholipid-PEG wrappings, following DSPE-PEG(5000) with 87.5%, whereas the relative surface coverages of the rest of the phospholipid-PEG molecules were <85% (Fig. 1c). However, there was no evident correlation between the relative surface coverage and the normalized response to fibrinogen (Fig. 2c). Even when looking at the absolute value of the normalized response versus the surface coverage, the correlation factor, which was found to be 0.26, was not statistically significant. Among the DNA wrapping, all the responses to the proteins were <30% intensity modulation. In comparison, a recently published work by our group showed that in response to small molecule neurotransmitters, DNA-wrapped SWCNT exhibited up to 80% intensity modulation^17^.

The discovery of a CoPhMoRe phase for fibrinogen has several practical implications (Supplementary Note 1), as it is one of the largest and most abundant plasma proteins, having a molecular weight of 340 kDa and normal concentrations^44^ of 1.75–4.30 g l^−1^. It has an elongated trinodular structure of 45–50 nm in length^31^,^32^, consisting of two outer globular domains (D-domains) of 6.5 nm in diameter and one central globular domain (E-domain) of 5.0 nm in diameter, which are connected by coiled coils of helical chains of 1.5 nm in diameter^45^.

We therefore hypothesize that both the corona phase specifically adopted by the DPPE-PEG(5000) upon adsorption onto the SWCNT surface and the unique conformation of fibrinogen, an elongated molecule of high aspect ratio, which is distinctive relative to the structure of the other proteins in the study (Supplementary Fig. 4), must be important factors in this molecular recognition. Moreover, the three-dimensional structure of fibrinogen enables multiple binding sites on the protein to be in close proximity to the SWCNT corona simultaneously, owing to the one-dimensional structure of the nanotube, as illustrated in Fig. 2d.

### SWCNT–fibrinogen interaction spectroscopy

**Figure 3:**
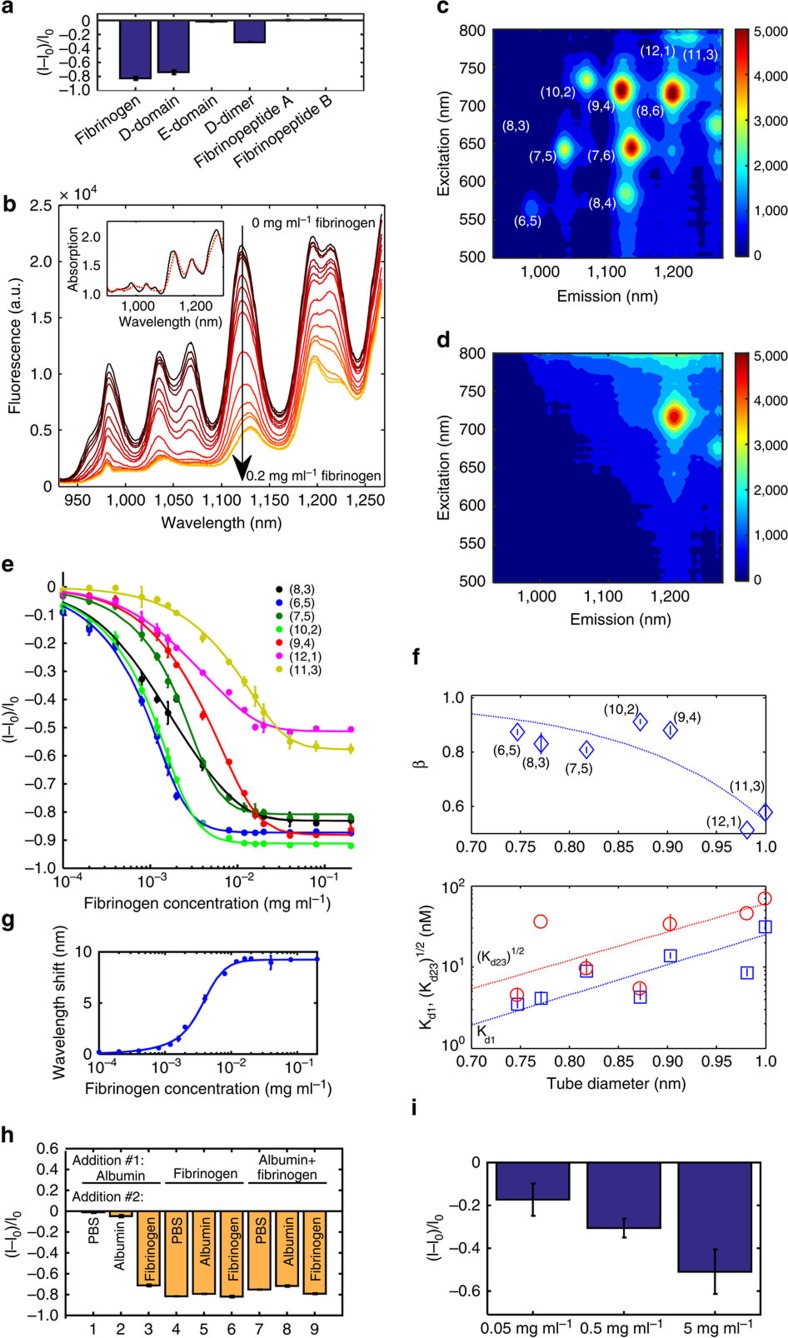
Fluorescence spectroscopy of the SWCNT–fibrinogen interaction. (**a**) Relative fluorescent response of DPPE-PEG(5000)-SWCNT (1 mg l^−1^) sensor to fibrinogen fragments (20 μg ml^−1^). (**b**) Fluorescent emission spectra of DPPE-PEG(5000)-SWCNT with 0, 10^−4^, 2 × 10^−4^, 4 × 10^−4^, 8 × 10^−4^, 1.2 × 10^−3^, 1.6 × 10^−3^, 2 × 10^−3^, 4 × 10^−3^, 8 × 10^−3^, 1.2 × 10^−2^, 1.6 × 10^−2^, 2 × 10^−2^, 4 × 10^−2^, 8 × 10^−2^ and 0.2 mg ml^−1^ fibrinogen show substantial decrease in emission intensity with increasing protein concentration. Inset: absorption spectra of DPPE-PEG(5000)-SWCNT suspension before (solid black curve) and after (dashed red curve) the addition of 0.02 mg ml^−1^ fibrinogen. (**c**) Excitation–emission profile of the DPPE-PEG(5000)-SWCNT solution before and (**d**) after the addition of 0.02 mg ml^−1^ fibrinogen. (**e**) The normalized fluorescent response of the various chiralities in the DPPE-PEG(5000)-SWCNT suspension to the addition of different concentrations of fibrinogen (dots). The fit according to the model described in the text is plotted as solid lines. (**f**) The parameters of the model used for data fitting in **d** and their 95% confidence intervals. Dashed lines are guides to the eye. Top panel: the proportional parameter *β* used to fit the normalized fluorescent response model. Bottom panel: the parameters *K*_d1_, and (*K*_d23_)^1/2^ used to fit the normalized fluorescent response model (blue squares and red circles, respectively). (**g**) Wavelength redshift of the (6,5) fluorescent emission peak of the DPPE-PEG(5000)-SWCNT suspension to the addition of different concentrations of fibrinogen (dots). The fit according to the model described in the text is plotted as a solid line. (**h**) Sensor performance in a complex environment: relative fluorescent response of DPPE-PEG(5000)-SWCNT suspension following a two-step analyte addition. First, either albumin (columns 1–3), fibrinogen (columns 4–6), or an equal mixture of both (columns 7–9) was added to the solution to a final concentration of 20 μg ml^−1^ and incubated for an hour. Then either PBS (columns 1, 4 and 7), albumin (columns 2, 5 and 8), or fibrinogen (columns 3, 6 and 9) was added to a final protein concentration of 40 μg ml^−1^. The fluorescent response was measured after an additional 1 h incubation. (**i**) Relative fluorescent response of DPPE-PEG(5000)-SWCNT (5 mg l^−1^) sensor to fibrinogen (0.05, 0.5 and 5 mg ml^−1^) in serum. Error bars represent the s.d. between three replicate experiments.

To elucidate the mechanism of fibrinogen binding, we tested the response of the DPPE-PEG(5000)-SWCNT sensor to various fibrinogen fragments, including the D-domain, E-domain, D-dimer, fibrinopeptide A and fibrinopeptide B (Fig. 3a). A significant decrease in fluorescent emission, comparable to the response induced by the full-length fibrinogen protein, was observed for the D-domain, while the D-dimer, which is comprised of two D-domains, showed a large response as well, but to a lesser extent. The E-domain and the two fibrinopeptides produced no response. We thus conclude that the two outer *D* regions of the fibrinogen are the driving force of the interaction between the whole protein and the DPPE-PEG(5000)-SWCNT complex. Owing to the evident affinity between the *D* region and the DPPE-PEG(5000)-SWCNT corona, we hypothesize that the underlying interaction mechanism is a three-step sequential binding initiated by one of the *D* regions, followed by the other *D* region, culminate in the binding of the middle *E* region, leading to the strong interaction between the full-length protein and the SWCNT scaffold. This is supported by previous studies showing multistage adsorption of fibrinogen onto surfaces^46^, where an initial binding is followed by the reorientation of the elongated fibrinogen molecule.

To support this mechanism, we examined the fibrinogen concentration-dependent interaction with the various SWCNT chiralities^47^. We have recorded the fluorescent spectra of the DPPE-PEG(5000)-SWCNT with increasing concentrations of the protein, clearly observing a gradual decrease of the emission intensity, and a redshift of the emission peaks (Fig. 3b). The SWCNT absorption peaks heights remain invariant to the addition of the fibrinogen, ruling out nanotube aggregation effects, while the absorption peak wavelength undergoes a modest redshift (Fig. 3b, inset), indicating an increase in the dielectric constant and higher water content in the close proximity of the nanotube surface^48^. The relative fluorescent response of the SWCNT with larger band gaps, corresponding to smaller diameters^49^, is more pronounced relative to the larger diameter nanotubes, manifested in the excitation–emission profiles of the SWCNT sample before (Fig. 3c) compared with after (Fig. 3d) the addition of fibrinogen.

The fluorescent emission spectra in Fig. 3b were deconvoluted to the various SWCNT chiralities within the sample^16^ (Supplementary Fig. 3) to analyse each chirality independently. The normalized fluorescent response of each nanotube species is plotted in Fig. 3e versus the fibrinogen concentration in the solution, yielding a calibration curve of the fibrinogen sensor. Following our three-step sequential binding hypothesis, we model the fibrinogen–DPPE-PEG(5000)-SWCNT interaction by the following reaction scheme^50^:


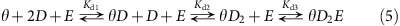


Where *θ* is an empty binding site on the SWCNT surface, *D* and *E* stand for the *D* and *E* regions of the fibrinogen, respectively, and *K*_d1_, *K*_d2_ and *K*_d3_ are the corresponding dissociation constants. To simplify the picture, we assume that upon the initial binding of the first *D* region, the fibrinogen molecule aligns with the SWCNT principle axis, and the remaining *D* and *E* regions bind instantaneously, allowing us to combine the last two steps of the reaction:


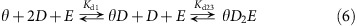


where *K*_d23_=*K*_d2_*K*_d3_. For further simplicity, we label the two types of the fibrinogen domains by *L*, which now stands for a single nodule of the protein molecule:


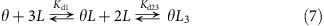


The total binding sites remain constant and thus fulfil the following equality:


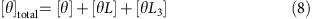


where the brackets represent concentration of the relevant variable.

The normalized fluorescent intensity response is linearly proportional to the relative number of bound sites on the SWCNT surface^16^:


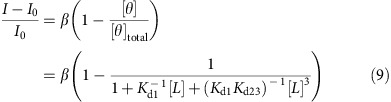


where *I*_0_ and *I* are the initial and final fluorescent intensity and *β* is the proportional factor. The fit to the data is plotted in Fig. 3e (solid lines), and the corresponding fit parameters, *β*, *K*_d1_ and (*K*_d23_)^1/2^ and their 95% confidence intervals are summarized in Fig. 3f (diamonds in the top panel, and blue squares and red circles in the bottom panel, respectively). Since (*K*_d23_)^1/2^ is larger than *K*_d1_, we can conclude that there is no accumulation of the intermediate reactants, implying that once the first nodule of fibrinogen is bound, the complete binding of rest of the molecule follows^50^.

On the basis of the deconvolution data, we plotted the wavelength redshift of the (6,5) fluorescent emission peak versus the fibrinogen concentration in the solution, yielding an additional calibration curve for the fibrinogen sensor (Fig. 3g). The advantage of calibrating based on the wavelength shift, as opposed to the intensity modulation, is that the units in the former case are not arbitrary and can be taken to be either the wavelength or the energy, eliminating the need for an internal standard or pre-calibration. The fit to the data points was achieved using the same model described above, yielding the fit parameters *K*_d1_=30.11 nM, and (*K*_d23_)^1/2^=7.41 nM, both within one order of magnitude of the fit parameters in the normalized intensity modulation calibration.

This observed solvatochromic shift corresponds to an increase of the dielectric constant in the close proximity of the SWCNT^36^ due to the adsorption of the fibrinogen molecules on the nanotubes' surface. Such an effect, explained by dielectric screening of repulsive Coulomb interactions^51^, has been shown to induce a decrease in fluorescent emission intensity^52^, and hence has an important role in the underlying quenching mechanism in this case. Moreover, a quenching effect of SWCNT fluorescent emission was previously reported for proteins adsorbed directly onto the surface of the nanotubes in the work of Barone *et al.*^53^, for example, where a significant decrease in emission intensity was observed on the exchange of a tightly packed SC wrapping with a glucose oxidase layer.

To assess the sensor performance in a complex environment, we tested its response to fibrinogen in the presence of albumin (Fig. 3h), which is the most abundant protein in the blood and constitutes about half of all the plasma proteins^54^. A DPPE-PEG(5000)-SWCNT suspension was first incubated with either albumin (Fig. 3h, columns 1–3), fibrinogen (columns 4–6), or an equal mixture of both proteins (columns 7–9), followed by the addition of just buffer (PBS, columns 1, 4 and 7), albumin (columns 2, 5 and 8), or fibrinogen (columns 3, 6 and 9). In contrast to the interaction with albumin, which produces little to no response (columns 1 and 2), the addition of fibrinogen results in a significant fluorescence decrease (columns 3–9), regardless of the order of the addition of the proteins. Further, we tested the response of the fibrinogen sensor in serum environment by adding fibrinogen solution to DPPE-PEG(5000)-SWCNT suspension in 10% fetal bovine serum (FBS) in PBS, to a final fibrinogen concentration of 0.05 mg ml^−1^, 0.5 mg ml^−1^ and 5 mg ml^−1^ (Fig. 3i and Supplementary Fig. 5a). The normal fibrinogen concentrations in human blood^44^ are between 1.75 and 4.3 mg ml^−1^, and are included in the tested range. The SWCNT sensor showed a significant signal quenching in response to fibrinogen of 17, 31 and 47% for the tested protein concentrations, respectively. Compared with the sensor response in PBS solution with similar conditions (90% quenching for 5 mg ml^−1^ protein concentration, Supplementary Fig. 5b), the response in serum is less pronounced, in agreement with previously published results^55^,^56^ showing that many serum components interact with SWCNT and can interfere with the interaction with the fibrinogen in this case.

An additional spectroscopy tool that can be used to determine the structure and conformation of proteins is circular dichroism. The circular dichroism spectrum of fibrinogen shows two peaks, at 209 and 220 nm (Supplementary Fig. 6, black solid curve), in agreement with previous findings^57^, and is typical to the mostly alpha helical structure^58^ found in fibrinogen^31^. The DPPE-PEG(5000)-SWCNT, however, has no characteristic circular dichroism signature^59^,^60^ except for mild absorption (Supplementary Fig. 6, dotted green curve). Comparing the circular dichroism spectra of fibrinogen with DPPE-PEG(5000)-SWCNT (Supplementary Fig. 6, dashed blue curve) to a pure fibrinogen solution, the positions of the two major peaks remain invariant, indicating that the SWCNT corona phase did not catalytically denature the fibrinogen protein^60^.

The Raman scattering spectrum of the DPPE-PEG(5000)-SWCNT also remains invariant to the interaction with fibrinogen, including the G-peak position and the G/D peak ratio (Supplementary Fig. 7a–c), assuring that there is no covalent interaction between the fibrinogen molecule and the carbon lattice of the nanotube, which keeps its *sp*^2^ hybridization^17^. The net surface charge of both the fibrinogen and DPPE-PEG(5000)-SWCNT does not change upon their interaction, shown by constant zeta potential values for all cases (Supplementary Fig. 7d).

### SWCNT–fibrinogen interaction dynamics

**Figure 4:**
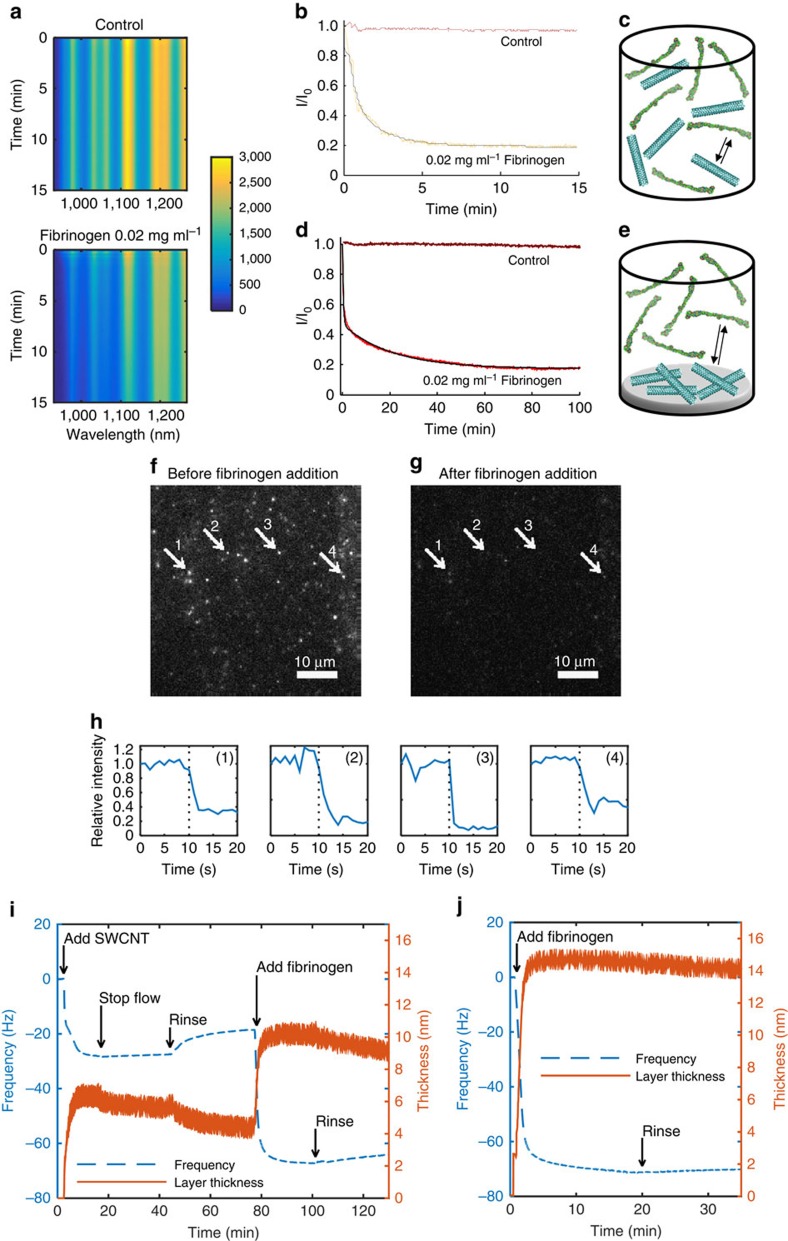
Dynamics of the SWCNT–fibrinogen interaction. (**a**) Fluorescent emission spectra of 1 mg l^−1^ DPPE-PEG(5000)-SWCNT, to which either PBS (top) or 0.02 mg ml^−1^ fibrinogen (bottom) was added 10 s after the beginning of the experiment. Since laser excitation was turned off while adding the solutions, the time point of the PBS or fibrinogen addition appears as a horizontal line with zero intensity. (**b**) The (10,2) chirality fluorescent emission peak over time of 1 mg l^−1^ DPPE-PEG(5000)-SWCNT, to which either PBS (control) or 0.02 mg ml^−1^ fibrinogen was added. Data taken from **a**. (**c**) Illustration of solution phase experiment. (**d**) Fluorescent emission over time of immobilized DPPE-PEG(5000)-SWCNT in agarose hydrogel, to which either PBS (control) or 0.02 mg ml^−1^ fibrinogen was added. (**e**) Illustration of hydrogel phase experiment. (**f**) Single DPPE-PEG(5000)-SWCNT fluorescence recorded by a 2D nIR camera before and (**g**) after the addition of fibrinogen. (**h**) Corresponding intensity time traces of the four diffraction limited single SWCNT spots marked in panels **f** and **g**. The dotted line represent the time point at which fibrinogen was added. (**i**) Frequency change (dashed blue curve) and layer thickness (solid orange curve) as measured by QCM-D, and calculated by the Voigt viscoelastic model, respectively, for a fibrinogen layer deposited on top of a DPPE-PEG(5000)-SWCNT layer, and for (**j**) fibrinogen layer deposited directly on top of the gold-coated quartz crystal.

The emission spectra of DPPE-PEG(5000)-SWCNT, to which either PBS or fibrinogen was added, were recorded every 2 s under continuous 785 nm laser excitation for 15 min (Fig. 4a). The fluorescent intensity of the DPPE-PEG(5000)-SWCNT suspension rapidly drops following the addition of fibrinogen, while the emission intensity of the control sample remains stable throughout the course of the experiment. This is manifested by tracking the (10,2) chirality peak, at 1,069 nm, in both cases (Fig. 4b).

To fit the dynamic data, we numerically integrated the rate equation resulting from our model in equation (9):


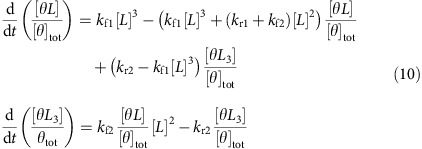


where *k*_f1_, *k*_f2_ and *k*_r1_, *k*_r2_ are the forward and the reverse rate constants of the first and second sequential steps in the association model, respectively, such that *K*_d1_=*k*_r1_/*k*_f1_ and *K*_d23_=*k*_r2_/*k*_f2_. The fit parameters resulted in *K*_d1_=39.7 nM and (*K*_d23_)^1/2^=42.7 nM, which is within one order of magnitude agreement with the dissociation contents of the (10,2) chirality peak calculated from the calibration curve fit (4.3 and 5.34 nM, respectively, Fig. 3e). A possible source for the difference is the additional data processing performed on the calibration data, which involved deconvoluting the spectra to find the individual fluorescent intensity contribution of the various nanotube chiralities in the sample.

Given that this set of experiments was done in solution phase as illustrated in Fig. 4c, one can speculate about the role that free DPPE-PEG(5000) polymer has in the interaction and signal transduction. To address this, we immobilized the DPPE-PEG(5000)-SWCNT on top of a thin agarose hydrogel bed^61^, followed by exhaustive washing of any unbound SWCNT or polymer molecules. The fluorescent emission peak of the (6,5) SWCNT chirality was monitored over 100 min, where both a control sample and a sample to which fibrinogen was added showed similar behaviour to the solution phase experiments, but on a longer time scale (Fig. 4d). This is expected based on the fact that only the fibrinogen molecules are mobile and can diffuse in the solution and the agarose hydrogel, while the SWCNT sensors are immobilized within the gel (Fig. 4e). Fitting the dynamic data with numerical integration of equation (10) results in *K*_d1_=49.8 nM and (*K*_d23_)^1/2^=33.7 nM, which is within approximately one order of magnitude agreement with the dissociation contents of the (6,5) chirality peak calculated from the calibration curve fit (3.5 and 4.4 nM, respectively, Fig. 3e). Similarly, a source for the difference is that the immobile sensor single channel detector integrates the fluorescent emission in the range of 950–1,050 nm, whereas the calibration spectra were recorded with a nIR spectrometer and were deconvoluted to the various SWCNT chiralities in the suspension.

Further experiments were conducted with surface-immobilized single SWCNT to conclusively demonstrate that nanoparticle aggregation is not responsible for the optical response. DPPE-PEG(5000)-SWCNT solution was deposited on a glass slide and was left to dry. Subsequently, the slide was extensively washed to remove any unbound nanotubes from the surface. The fluorescence of the individual nanotubes was imaged continuously using a 2D nIR camera attached to an inverted microscope, allowing the visualization of diffraction limited spots of single nanotubes (Fig. 4f and Supplementary Movie). Following the addition of fibrinogen, the fluorescent emission intensity significantly decreases (Fig. 4g), as evident from the intensity traces over time (Fig. 4h). These results support the claim that the mechanism of emission intensity decrease does not involve nanotubes aggregation effects, since a similar decrease is observed when the SWCNT are either in solution phase, or immobilized within a hydrogel or on a glass slide, where they cannot aggregate.

The gradual adsorption of the fibrinogen proteins to the DPPE-PEG(5000)-SWCNT was further verified by QCM-D measurements. The QCM-D instrument (Q-sense E4) was used to gain additional insight on this process by monitoring the adsorption of the fibrinogen proteins onto a nanotube layer deposited on a gold-coated crystal and calculating the resulting layers' thickness. Both of the adsorption steps demonstrated a gradual decrease in the frequency of the oscillating crystal, indicating a gradual increase in the layer thickness (Fig. 4i). The thickness of the SWCNT bottom layer was estimated as 4.4±0.1 nm, and the total thickness of both the SWCNT and the fibrinogen layers was estimated as 9.4±0.2 nm, yielding a protein layer of ∼5 nm in thickness. In the case of SWCNT, the PBS wash resulted in the loss of ∼20% of the layer thickness, whereas in the fibrinogen layer, the rinse resulted in the removal of ∼25% of the layer. In contrast, when the fibrinogen solution was added directly to a gold-coated quartz crystal surface, the final thickness of the protein layer was 14.2±0.1 nm (Fig. 4j), which is more than twice the thickness of the fibrinogen layer formed on top of the SWCNT layer. Previous studies of fibrinogen adsorption onto a gold surface using QCM-D technique have shown that the fibrinogen proteins form a monolayer on the surface^62^, in agreement with the widely accepted assumption of a monolayer surface-adsorption of proteins in general^63^. Taking into account the physical dimensions of the protein, our findings imply that the fibrinogen molecules mostly lay horizontally flat (side-on) when adsorbed onto the DPPE-PEG(5000)-SWCNT, whereas when adsorbed directly onto the gold surface, they can adopt different configurations, including an end-on adsorption with the other unbound end sticking out into the aqueous solution. Previous studies of fibrinogen adoption onto various surfaces for haemocompatibility evaluation have reported monolayer thickness ranging between 2 and 37 nm, depending on the surface properties, where thickness values above 10 nm indicated an end-on adsorption^64^,^65^.

### SWCNT–fibrinogen interaction microscopy

**Figure 5:**
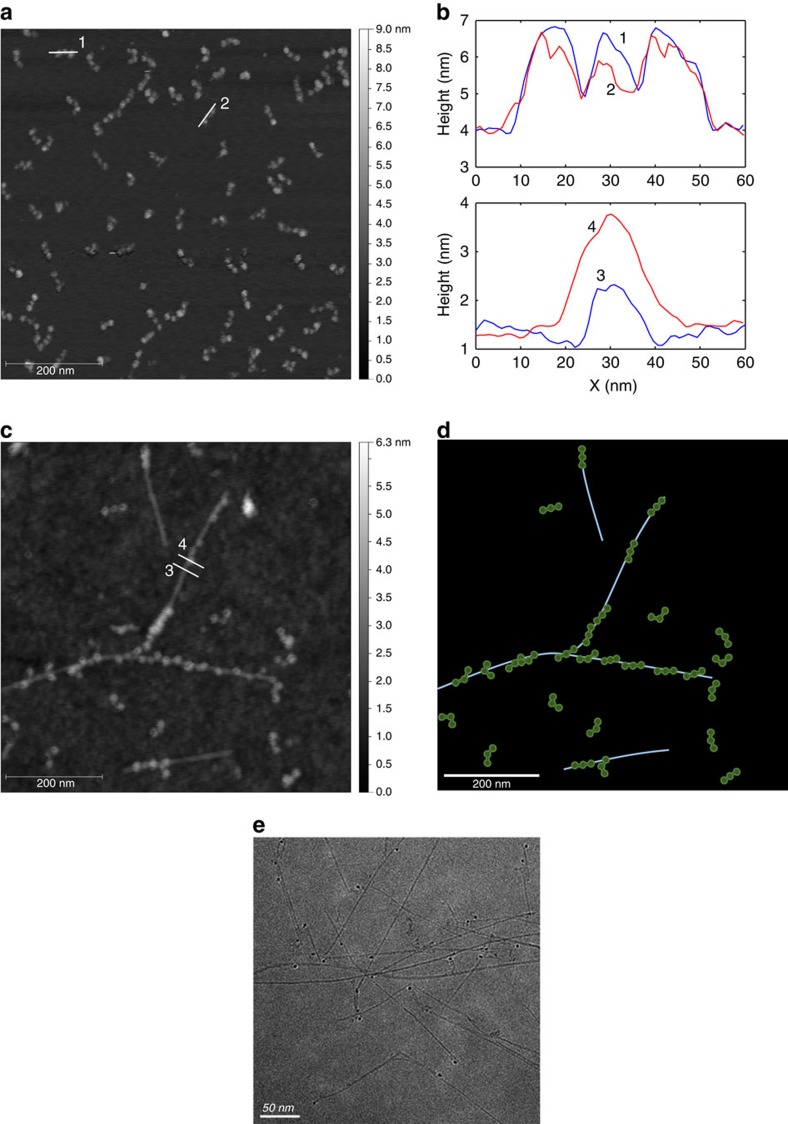
High resolution microscopy of the SWCNT–fibrinogen interaction. (**a**) AFM image of fibrinogen molecules on MICA surface. Scale bar, 200 nm. (**b**) Height profiles along the lines in the AFM images. (**c**) AFM of DPPE-PEG(5000)-SWCNT with fibrinogen on a silicon wafer. Scale bar, 200 nm. (**d**) Illustration of the SWCNT (blue lines) and fibrinogen molecules (green dumbbells) in panel **c**. (**e**) Cryo-TEM image of DPPE-PEG(5000)-SWCNT with fibrinogen, showing individually suspended SWCNT and no bundling. The dark spheres are catalyst particles. Scale bar, 50 nm.

Fibrinogen molecules were imaged on a mica surface with tapping mode AFM, clearly showing the unique dumbbell structure of the fibrinogen (Fig. 5a). The fibrinogen molecules were scattered uniformly on the surface, randomly orientated, with each molecule being composed of three small spherical nodes. The height profiles along the principle axis of the fibrinogen (labelled as 1 and 2 in Fig. 5a) show characteristic three-peak traces (Fig. 5b, top panel), as expected from the tri-nodule structure of the protein and in agreement with previous findings^31^.

Following incubation with DPPE-PEG(5000)-SWCNT, most of the fibrinogen molecules appear to be bound to SWCNT (Fig. 5c), demonstrating the physical interaction between the two. Comparing the height profiles across a bare SWCNT and across a fibrinogen molecule that is bound to SWCNT (labelled as 3 and 4 in Fig. 5c), we can conclude that the fibrinogen molecule is stacked on top of the nanotube (Fig. 5b, bottom panel). The inter-molecular distance between the adsorbed fibrinogen molecules is 22±20 nm, resulting in an average of 8 to 9 proteins per SWCNT. Furthermore, the fibrinogen orientation does not appear to be isotropically distributed, but rather the protein molecules seem to align along the axis of the nanotubes. In Fig. 5d, a schematic of the adsorbed fibrinogen constructed from the experimental AFM positions and orientations underscores this observation. This further supports the layer thickness of fibrinogen adsorbed onto SCWNT calculated from the QCM-D data, which indicate a monolayer adsorption of the protein molecules laying horizontally flat (side-on).

Cryo-TEM captures solution samples in their native hydrated environment and avoids artifacts of sample drying^66^. A sample of DPPE-PEG(5000)-SWCNT incubated with fibrinogen was rapidly frozen in liquid nitrogen and imaged with cryo-TEM. The resulting images show that the SWCNT remain individually suspended in the solution, and no aggregation occurs following the interaction with the protein (Fig. 5e). This finding additionally confirms that nanoparticle aggregation is not the mechanism of the fibrinogen optical response.

## Discussion

We have demonstrated protein CoPhMoRe for the first time. By screening the response of the fluorescent emission of a library of DNA, RNA and phospholipid-PEG suspended SWCNT following the interaction with a library of human proteins, we discovered that the DPPE-PEG(5000)-SWCNT complex acts as a selective sensor for fibrinogen.

Although DPPE-PEG(5000) had one of the highest relative SWCNT surface coverages compared with various PEGylated lipids, DNA or RNA wrappings, no correlation between surface coverage and recognition was found. Moreover, the selective response is not explained by elementary protein parameters such as molecular weight, isoelectric point or relative surface hydrophobicity, whereas circular dichroism spectroscopy rules out protein denaturation. Lastly, the invariant height and width of the absorption peaks, and the individually suspended SWCNT imaged by cryo-TEM, rule out nanotube aggregation or precipitation as the fluorescent quenching mechanism, supported by the successful demonstration of the sensor response when immobilized within a hydrogel or on a glass surface. Hence, we conclude that the specific corona phase formed by the DPPE-PEG(5000) enables recognition of the unique tri-nodule fibrinogen protein.

A detailed analysis of this interaction reveals a mechanism of sequential binding of the three fibrinogen nodules, starting with one of the outer *D* regions binding to the SWCNT, followed by the other *D* and *E* regions, and the alignment of the protein along the nanotube, resulting in the complete binding of the three fibrinogen nodes to the SWCNT surface and a substantial fluorescent emission quenching. This reaction mechanism is supported by fitting kinetic experimental results and high-resolution AFM. Moreover, QCM-D measurements show the monolayer adsorption of fibrinogen onto surface-deposited DPPE-PEG(5000)-SWCNT, forming a thin layer whose thickness indicates that the protein molecules lay horizontally flat on the underlying nanotubes.

The DPPE-PEG(5000)-SWCNT sensor responds to fibrinogen in the presence of albumin, which is the most abundant protein in blood and usually used as the gold standard in assessing non-specific protein adsorption, thus paving the way to use the sensor in a controlled environment with additional components. Furthermore, the sensor can detect fibrinogen in serum environment over a concentration range covering the clinically relevant concentrations of fibrinogen in human blood^44^ (1.75–4.3 g l^−1^). Finally, the ability of the sensor to detect the fibrinogen protein when immobilized within agarose gel opens the possibility of use *in vivo* with an implantable biocompatible hydrogel^15^.

In summary, we have demonstrated a molecular recognition motif, based on the corona phase of fluorescent nanoparticles, for the detection and quantification of fibrinogen, an important protein biomarker that is an essential factor in the blood coagulation cascade and the principal element of a thrombus. Our work motivates the search for novel CoPhMoRe phases for protein recognition. This methodology can generate new synthetic non-biological antibodies and provide an alternative for conventional antibodies, which suffer from major limitations including the need of a living organism for initial production, long development times, high production costs and challenging reproducibility, poor stability due to hydrolysis in ambient temperature and moderate acidic conditions resulting in limited shelf life, and sensitivity to degradation while circulating *in vivo*. In contrast, our SWCNT template recognition overcomes these shortcomings by offering a stable and reproducible construct that can push forward discovery research in the field allowing multiplexed high-throughput synthesis and library screenings.

## Methods

### DNA and RNA SWCNT suspension

Raw HiPCO (Unidym, 0.8–1.2 nm in diameter with 1 nm mean diameter and 100 nm—1 μm initial length) were processed by organic–aqueous phase separation followed by drying and homogenizing, as previously described^37^. DNA or RNA (2 mg) (Integrated DNA Technologies, Inc.) were added to 1 mg of SWCNT in 1 ml of 0.1 M sodium chloride solution, and sonicated while in an ice bath with 3 mm probe tip (Cole Parmer) for 40 min at a power of 4 W. Subsequently, samples were bench-top centrifuged (Eppendorf) for 180 min at 16,100 relative centrifugal force (RCF). The top 80% of the supernatant was carefully collected for further experimenting and the pellet was discarded. Successful suspensions were validated by recording their ultraviolet–visible-nIR absorption spectra (Shimadzu UV-3101PC).

Single-stranded DNA sequences used in this study were (GT)_15_, (AT)_30_, (GC)_30_, (AT)_15_, (AAAT)_7_, (ATTT)_7_ (GGGT)_7_, (GTTT)_7_, and the single-stranded RNA sequence used was (GU)_15_.

### Phospholipid-PEG SWCNT suspension

SWCNT were first suspended in SC, which was later removed by dialysis in the presence of phospholipid-poly(ethylene glycol) (phospholipid-PEG, Avanti Polar Lipids) as previously published^16^. Briefly, 40 mg of SWCNT was added to 2 wt% SC solution to a final SWCNT concentration of 1 mg ml^−1^and was bath sonicated (Branson 2510) for 10 min, followed by 6 mm probe tip sonication for 60 min at a power of 12 W while in an ice bath. The resulting dark solution was ultracentrifuged in a SW32 Ti rotor (Beckman Coulter) at 150,000 RCF for 4 h to remove SWCNT aggregates and impurities. The top 80% of the supernatant was carefully collected for further experimenting and the pellet was discarded (see Supplementary Fig. 8 for scanning electron microscopy image of the SC-SWCNT suspension). The mean nanotube length following the initial processing was 550 nm (ref. 16).

A 5 mg ml^−1^ solution of phospholipid-PEG in water was bath sonicated for 5 min to ensure complete dissolution. The phospholipid-PEG was diluted to a final concentration of 2 mg ml^−1^ in 40 mg l^−1^ of SC-SWCNT suspension and the mixture was dialysed using 1 kDa molecular weight cutoff dialysis cartridge against water for a period of 4 to 5 days with multiple water changes to ensure the complete removal of SC from the SWCNT surface, allowing for the adsorption of the phospholipid-PEG instead.

Successful suspensions were validated by recording their ultraviolet–visible-nIR absorption spectra (Shimadzu UV-3101PC) in a 1-cm path length quartz cuvette (Starma). The absorption spectra showed a redshift relative to the initial SC-SWCNT suspension (Supplementary Fig. 9), indicating the surfactant exchange^18^.

Phospholipid-PEG used in this study were DPPE-PEG(5000), DMPE-PEG(5000), DSPE-PEG(5000), DSPE-PEG(2000), DSPE-PEG(2000)-cyanur, DSPE-PEG(2000)-CA, DSPE-PEG(2000)-maleimide, DSPE-PEG(2000)-PDP, DSPE-PEG(2000)-amine, DSPE-PEG(2000)-biotin, and DSPE-PEG(350), where DPPE, DMPE and DSPE stand for dipalmitoyl-, dimyristoyl-, and distearoyl-phosphatidylethanolamine, respectively, CA denotes carboxylic acid and PDP denotes 3-(2-pyridyldithio)-propionyl (Fig. 1a). The number in parentheses is the molecular weight of the PEG chain in Daltons.

### High-throughput nIR photoluminescence screening

Human proteins were purchased from Sigma-Aldrich (USA) and were handled according to the supplier instructions. SWCNT suspensions were diluted in PBS to 1 mg l^−1^ concentration (0.036 absorption at 632 nm (ref. 16)) and aliquots were added to a 96-well plate followed by the addition of 2 vol% of the test protein analytes to a final protein concentration of 20 μg ml^−1^. Following 1 h incubation on a tabletop orbital shaker, the fluorescent spectra of the SWCNT were recorded using a custom-made nIR microscope array. Briefly, the 96-well plate was placed on top of a motorized stage of a Zeiss AxioVision inverted microscope, and the samples were excited by a 785-nm photodiode laser (B&W Tek Inc.) with 80 mW at the sample plane focused by × 20 objective for 10 s exposure time. The resulting fluorescent light was collected by a coupled nitrogen-cooled Princeton Instruments InGaAs 1D detector through a PI Acton SP2500 spectrometer (Supplementary Fig. 10a).

For testing the fluorescence response in serum, the SWCNT suspension was diluted in FBS (10 vol% in PBS) to 5 mg l^−1^ concentration. Samples of 100 μl fibrinogen solution in PBS were added to 100 μl of SWCNT in FBS, in a 96-well plate, to a final protein concentration of 0.05, 0.5 and 5 mg ml^−1^. Following 1 h incubation, the fluorescent spectra of the SWCNT were recorded using the custom-made nIR microscope array, as described above.

### Excitation–emission profile

A white light source coupled to a monochromator was used for the excitation of SWCNT samples that were placed on the stage of the nIR array described above (Supplementary Fig. 10a). The corresponding fluorescence of the sample was collected for excitation wavelength range of 400–800 nm in 5 nm steps with 90 s exposure time.

### Zeta potential

Samples were measured in a zeta potential analyzer (Zeta PALS, Brookhaven Instruments Corporation) with 10 runs of 20 cycles each, following 1 h incubation. All samples were kept in PBS at pH 7.4.

### Circular dichroism

Samples were analysed, following 1 h incubation, in a circular dichroism spectrometer (Aviv Model 202) in the wavelength range of 190–260 nm in 1 nm intervals, using a 1-mm path length quartz cuvette (Hellma). Signal from a reference sample of the PBS buffer was subtracted from the results.

### Raman spectroscopy

Raman spectra were acquired with a confocal Raman spectrometer HR-800 (Horiba JY) using a 633-nm laser source focused with a × 10 objective on the sample plane. Light was collected for 10 s with five accumulations. Samples of 300 μl fibrinogen and SWCNT in PBS were measured in a 96-well plate on top of the microscope stage, following 1 h incubation.

### Continuous fluorescent emission monitoring

Fluorescent emission was collected using the custom-made nIR microscope array as described above, with continuous laser excitation of a sample within a 96-well plate, and spectra acquisition every 2 s. The fibrinogen was added to the SWCNT solution while on the microscope stage during a 10-s break in laser illumination using the microscope port shutter. The shutter was opened immediately after analyte addition enabling laser excitation resumption.

### Immobilized sensors fluorescent monitoring

Agarose solution (0.2 wt% in water) was heated on top of a hot stirring plate until completely dissolved. The resulting clear solution was allowed to cool for 10 min, following which 50 μl aliquots were cast into the wells of a 96-well plate that was kept in a humidified environment for 30 min for the solution to gel. SWCNT solution (20 μl of 20 mg l^−1^) was spotted on top of each gel and the plate was placed in a humidified incubator at 37 °C for 30 min to allow for the incorporation of the SWCNT within the top layer of the agarose gel that was partially melted. Subsequently, the gels were washed with PBS to remove any unbound materials, and the well-plate was kept in a humidified environment at room temperature for additional 30 min for cooling and equilibrating before testing.

The fluorescent emission of the immobilized SWCNT on top of the agarose hydrogel was analysed using a custom-made portable nIR detector. Briefly, high-power 565-nm light-emitting diode was focused on the sample, and the emitted fluorescence was collected by a single channel InGaAs detector (Thorlabs) through a 900-nm long-pass dichroic mirror, followed by a 1,050-nm short pass filter, and a 950-nm long pass filter (Supplementary Fig. 10b), which isolated the fluorescent emission of the (6,5) SWCNT chirality.

### 2D nIR fluorescence microscopy

Single SWCNT fluorescence data were collected by a Zeiss AxioVision inverted microscope coupled to a nitrogen-cooled InGaAs 2D detector (Princeton Instruments), with 250 mW, 785 nm, laser excitation (Invictus, Kaiser Optical Systems), through × 100 objective (Zeiss, Apochromat, oil immersion). For surface immobilization of single SWCNT, a droplet of 50 μl of 1 mg l^−1^ SWCNT solution was deposited on a microscope slide and left to dry. Subsequently, the slide was extensively washed with PBS to remove any unbound nanotubes. The slide was placed on top of the microscope stage and imaged continuously with 1 s acquisition time. Fibrinogen was added by carefully dropping 20 μl of 0.1 mg ml^−1^ on top of the glass slide, without changing the imaging focal plane.

### Quartz crystal microbalance with dissipation

QCM-D measurements were conducted with a Q-Sense E4 instrument (Q-Sense, Sweden) on a gold-coated quartz crystal substrate mounted within a flow module. Both frequency and dissipation responses of six overtones of the crystal were monitored throughout the experiment. All the experiments were conducted at a fixed temperature of 25 °C. The crystal was first equilibrated under continuous flow of PBS (150 μl min^−1^) before introducing the SWCNT suspension (5 mg l^−1^ in PBS, 150 μl min^−1^), which was then allowed to adsorb under no flow. Afterwards, the flow cell was washed with PBS to remove unbound material. Subsequently, the protein solution (0.1 mg ml^−1^ in PBS, 150 μl min^−1^) was introduced into the flow chamber, followed by PBS wash. In a control experiment, only the protein solution was introduced into the flow cell and washed with PBS afterwards. Owing to the large dissipation response, indicating that the adsorbed layers do not behave as rigid materials^67^, the data were fitted by the viscoelastic-Voigt model for the SWCNT and the protein layers^68^ in two steps with the QTools software (Q-Sense, Sweden), to calculate the layers' thickness over time. The density of the SWCNT layer was estimated as 1.6 g cm^−3^ according to the manufacture (Unidym), whereas the density of the protein layer^69^ was taken to be 1.3 g cm^−3^.

### Atomic force microscopy

Silicon wafers were washed by isopropanol and water, and were blown dry by a nitrogen gun. Samples of 147 μl of 1 mg l^−1^ SWCNT were mixed with 3 μl of 0.1 mg ml^−1^ fibrinogen in PBS, and following 1 h incubation, 40 μl of the mixture was pipetted onto the wafer. In addition, 40 μl of 0.1 mg ml^−1^ fibrinogen in PBS was pipetted on top of a freshly cleaved mica (Grade V-1 Muscovite, SPI Supplies, Structure Probe, Inc.). The substrates were allowed to dry for 10 min in the fume hood and were washed again with water before imaging with Asylum Research MFP-3D AFM in tapping mode.

### Hydrophobicity maps

The three-dimensional structure of the proteins was visualized with Pymol Molecular Graphics System software (Version 1.7.1.3, Schrodinger LLC), using the corresponding protein data bank files (Supplementary Note 2). The hydrophobicity was visualized by a colour map from white (hydrophilic) to red (hydrophobic), according to a normalized scale^70^. The hydrophobic surface area was obtained by calculating the solvent accessible surface area of the hydrophobic amino acids relative to the total area.

### Cryo-TEM

A sample droplet (4 μl) was placed on top of a support film (lacey formvar/carbon on 200 mesh grid), which was then mounted on a Gatan 626 cryo-holder. The specimen was cooled by liquid nitrogen and was subsequently loaded onto a high-resolution analytical cryo-TEM (JEOL 2100 FRG) for imaging, operating at acceleration voltage of 200 kV, with magnification range between 10,000 and 60,000. Images were recorded with a Gatan 2 × 2 k UltraScan charge-coupled device camera.

### Scanning electron microscopy

A droplet of 20 μl of 10 mg l^−1^ SC-SWCNT was placed on top of an aluminium slab and was left in a fume hood to dry overnight. The sample was imaged with JEOL 6700F scanning electron microscope, using 5 kV accelerating voltage and the secondary electrons detector.

## Additional information

**How to cite this article:** Bisker, G. *et al.* Protein-targeted corona phase molecular recognition. *Nat. Commun.* 7:10241 doi: 10.1038/ncomms10241 (2016).

## Supplementary Material

Supplementary Information Supplementary Figures 1-10, Supplementary Table 1, Supplementary Notes 1-2 and Supplementary References

Supplementary Movie 1 Fibrinogen detection with single nanotube sensors
