# Supplementary material for: Protein-targeted corona phase molecular recognition
Source: Nat Commun. 2016 Jan 8;7:10241. doi: 10.1038/ncomms10241 (PMC4729864; doi:10.1038/ncomms10241)
Supplement: Supplementary Information — Supplementary Figures 1-10, Supplementary Table 1, Supplementary Notes 1-2 and Supplementary References [file ncomms10241-s1.pdf]

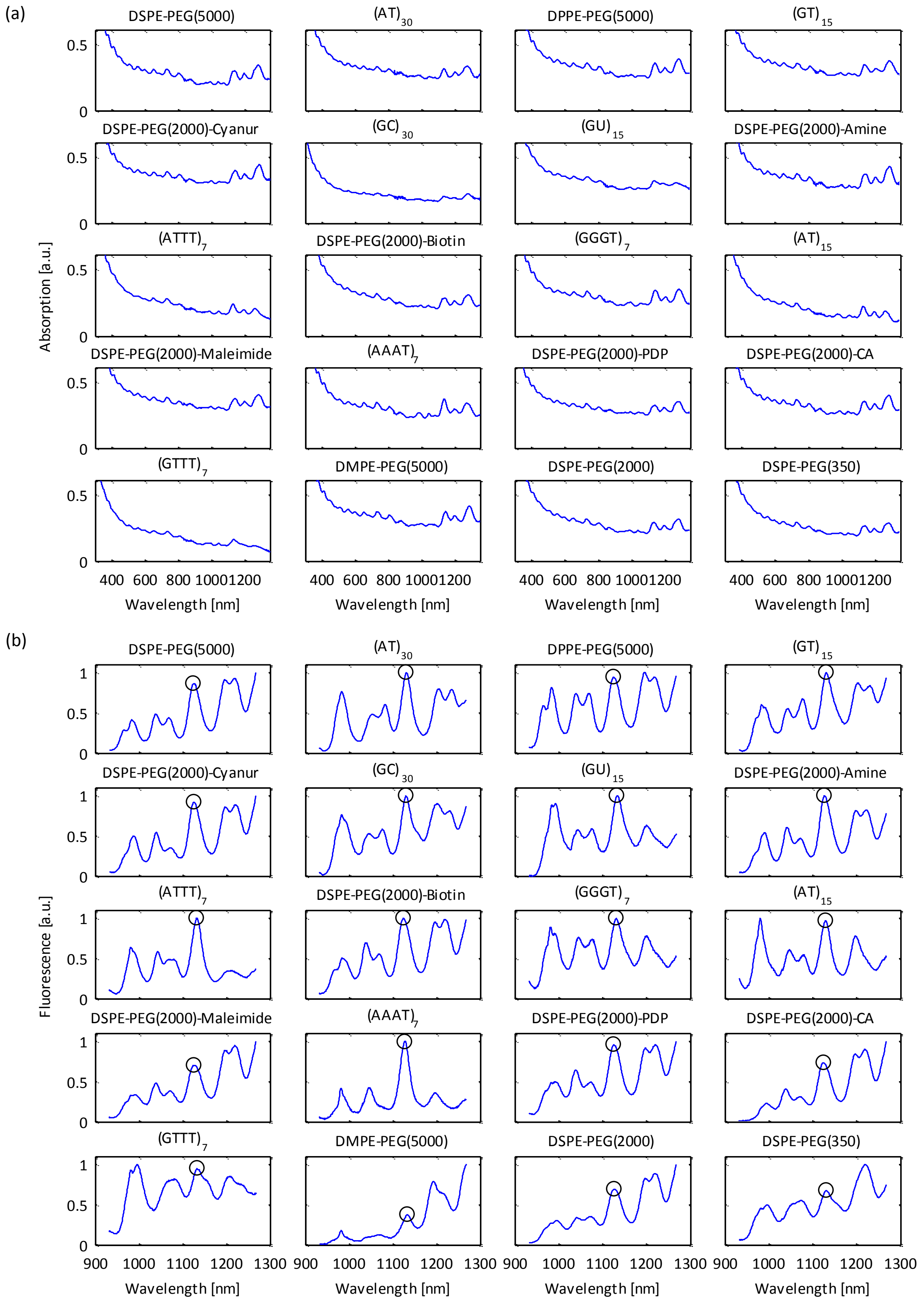

Supplementary Figure 1. SWCNT library. (a) Absorption spectra of the SWCNT suspensions. (b) Fluorescent emission spectra of SWCNT suspensions excited by a 785 nm laser. The joint peak of the (9,4) and the (7,6) chiralities is labeled with a circle.

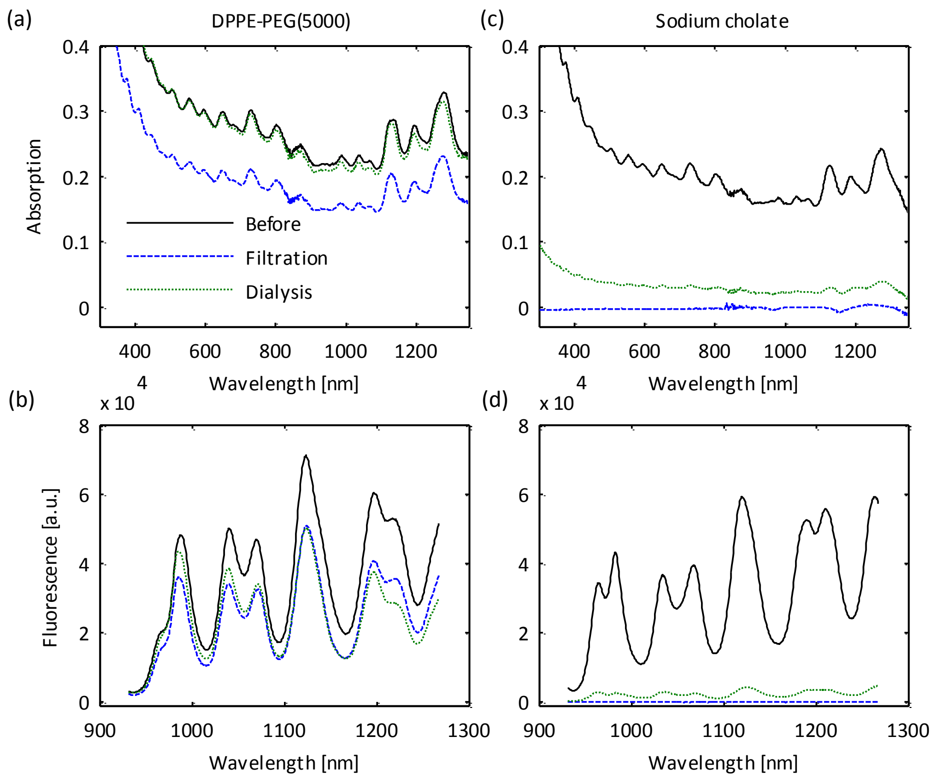

Supplementary Figure 2. SWCNT suspension stability. (a) Absorption and (b) fluorescence spectra of DPPE-PEG(5000) – SWCNT suspensions, before (solid black curves) and after the removal of excess phospholipid-PEG molecules either by filtration through a 100 kDa molecular weight cut off filter (blue dashed line), or by dialysis with 100 kDa molecular weight cut off membrane (dotted green curve). (c) Absorption and (d) fluorescence spectra of sodium cholate (SC) – SWCNT suspensions before and after the removal of excess SC molecules in a similar mannar. The DPPE-PEG(5000) – SWCNT suspension remains stable, whereas the SC sample aggregates and loses its colloidal stability as evident from the loss of both the absorption and fluorescent peaks.

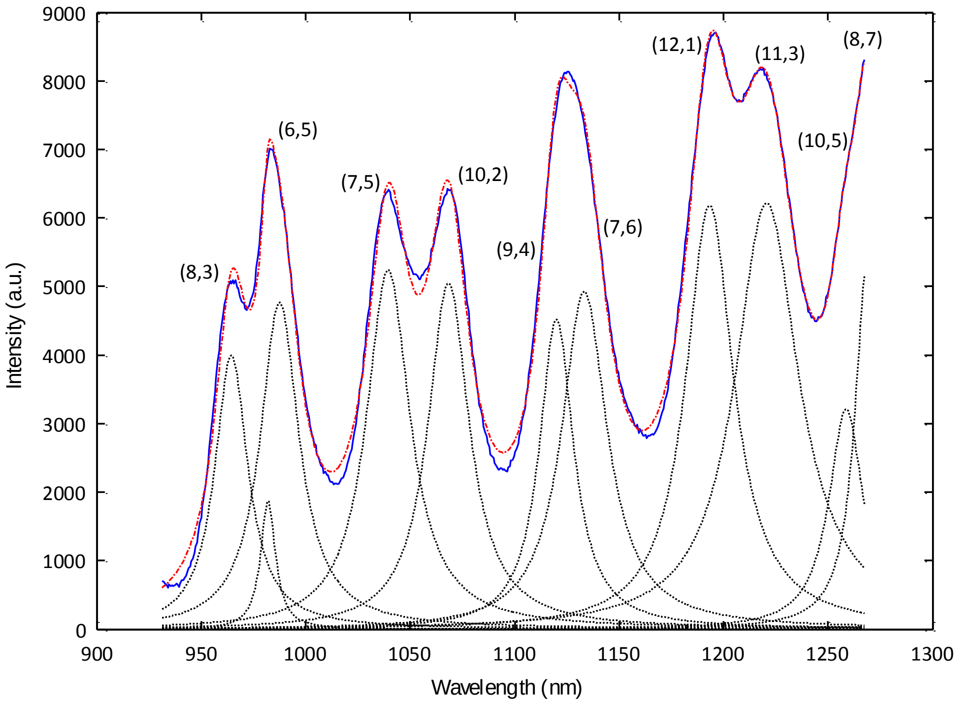

Supplementary Figure 3. Spectra deconvolution. SWCNT fluorescent emission spectrum (solid blue curve) and its deconvolution to the various chiralities (dashed black curves). The fit is illustrated by the dashed red curve.

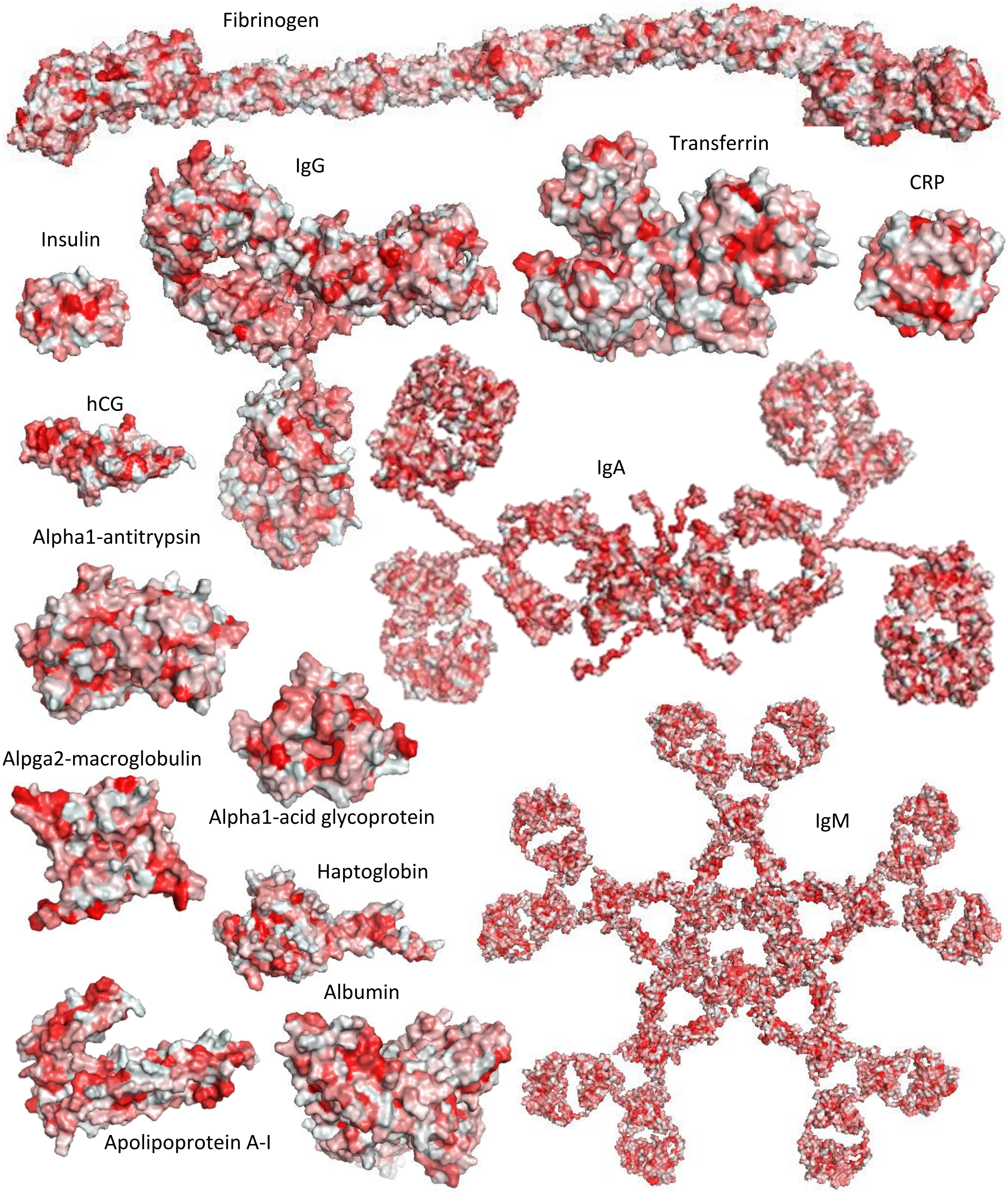

Supplementary Figure 4. 3D visualization of the proteins panel. The proteins surface is colored according to the hydrophobicity of each amino acid, where the color red is the most hydrophobic, and white is hydrophilic.

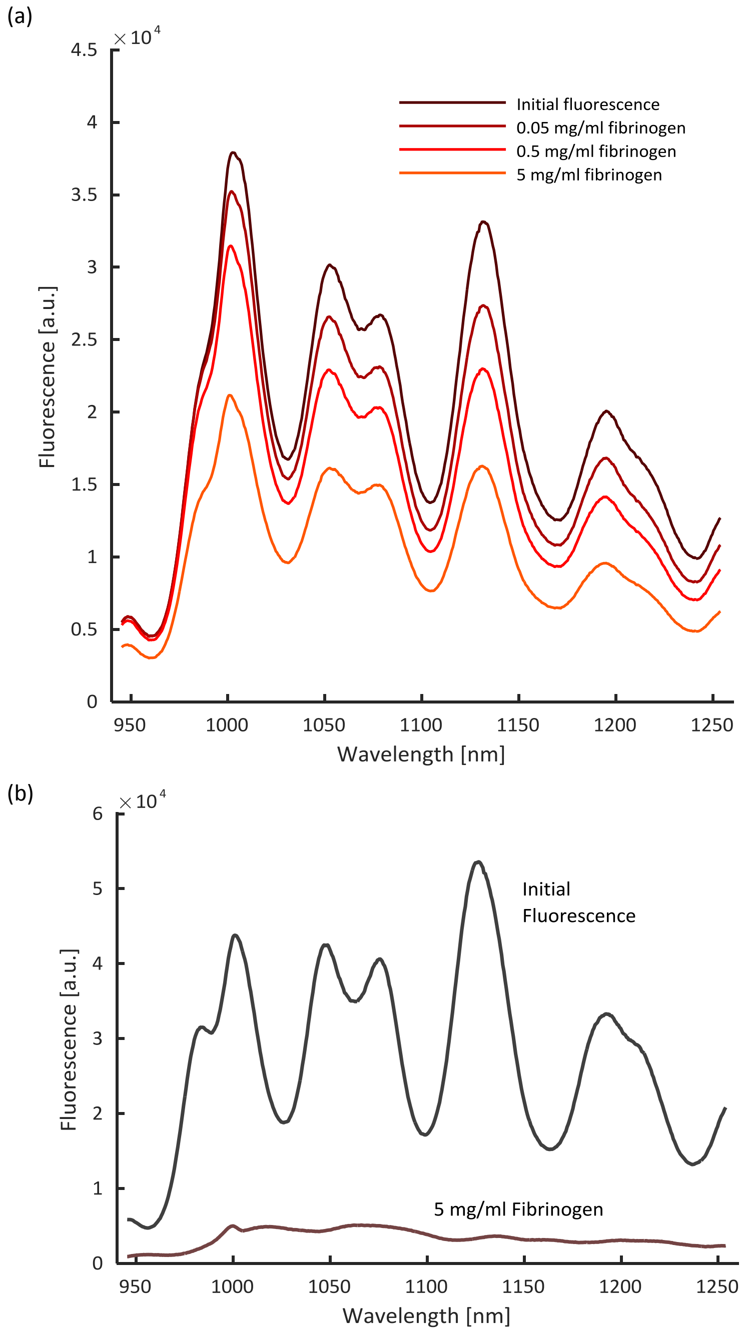

Supplementary Figure 5. Sensor functionality in serum environment. (a) Fluorescent response of DPPE-PEG(5000)-SWCNT suspension (5 mg L^{-1}) to fibrinogen (0.05, 0.5, and 5 mg ml^{-1}) in serum environment (10% fetal Bovine Serum in PBS). (b) Fluorescent response of DPPE-PEG(5000)-SWCNT suspension (5 mg L^{-1}) to fibrinogen (5 mg ml^{-1}) in PBS.

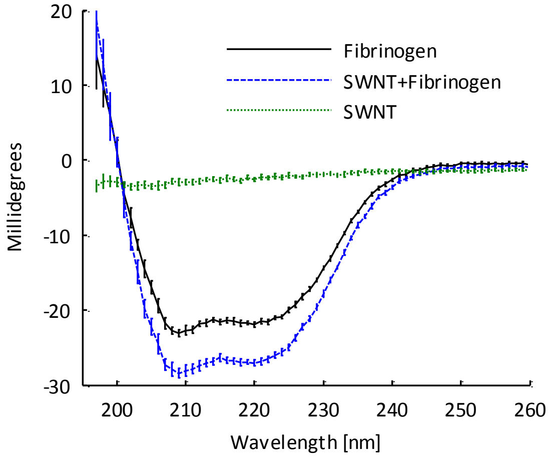

Supplementary Figure 6. Circular dichroism spectroscopy. Circular dichroism spectra of fibrinogen (0.02 mg ml^{-1}, black solid curve), DPPE-PEG(5000)-SWCNT (1 mg L^{-1}) with 0.02 mg ml^{-1} fibrinogen (blue dashed curve), and DPPE-PEG(5000)-SWCNT (1 mg L^{-1}, dotted green curve).

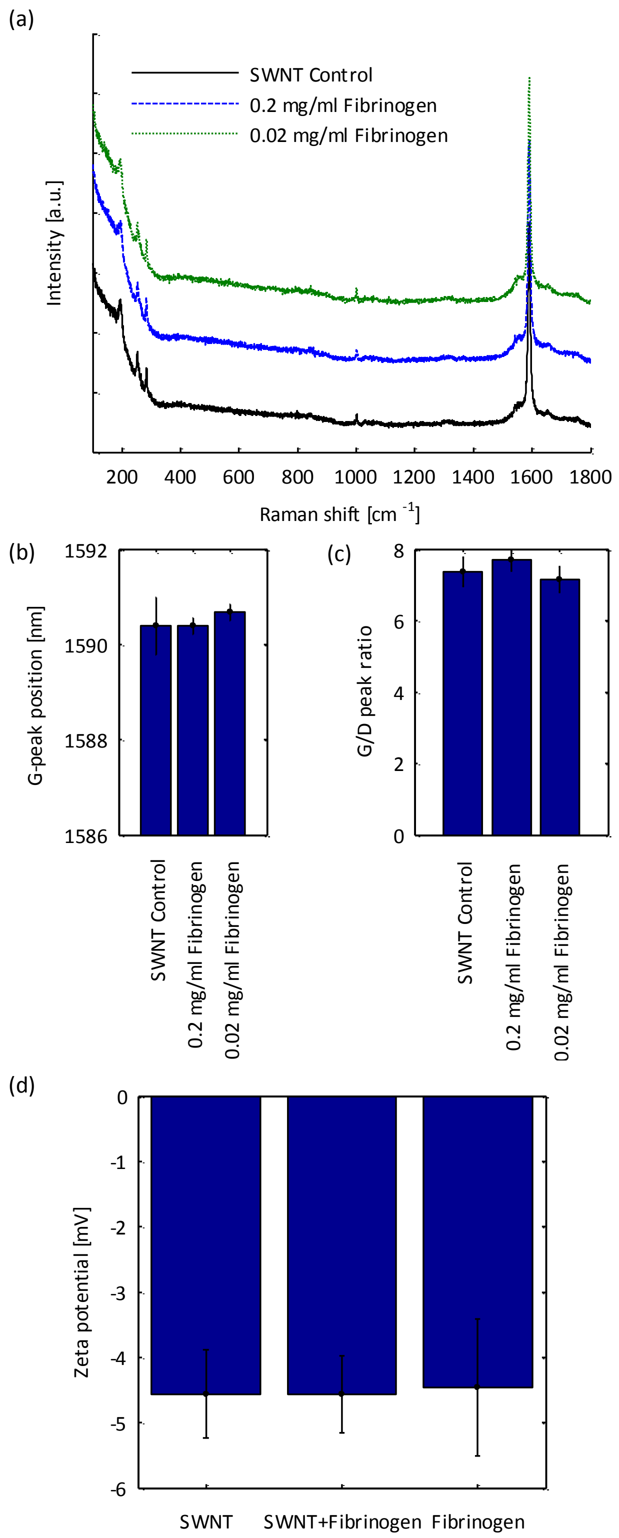

Supplementary Figure 7. Raman spectroscopy and zeta potential. (a) Raman spectra of DPPE-PEG(5000)-SWCNT (solid black curve), and of DPPE-PEG(5000)-SWCNT with 0.2 mg ml^{-1} (blue dashed curve) or 0.02 mg ml^{-1} (dotted green curve) fibrinogen. (b) G-peak position of each of the spectra in (a). (c) G/D peak ratio of each of the spectra in (a). (d) Zeta potential of DPPE-PEG(5000)-SWCNT, fibrinogen, and DPPE-PEG(5000)-SWCNT with fibrinogen.

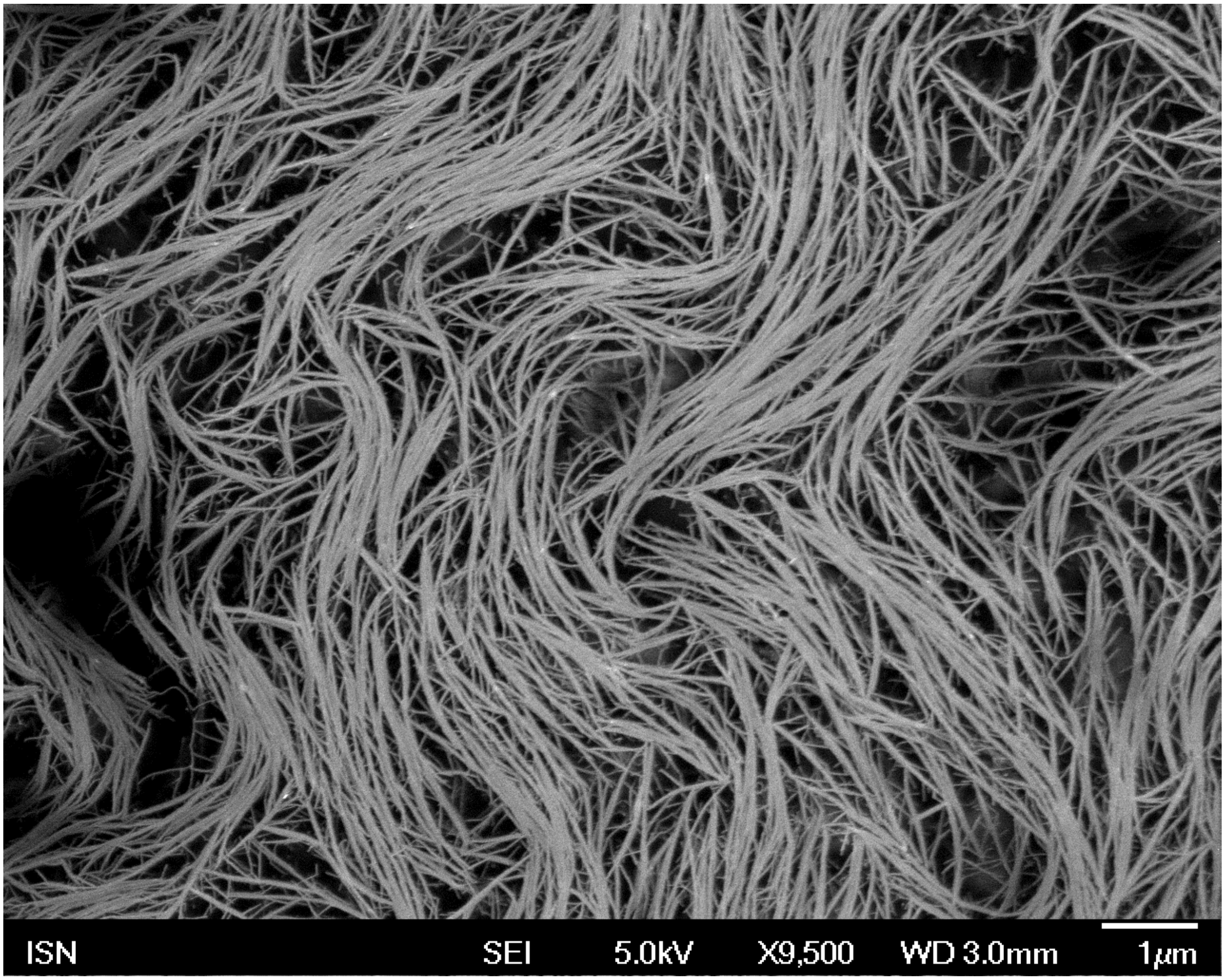

Supplementary Figure 8. SEM image of SC-SWCNT.

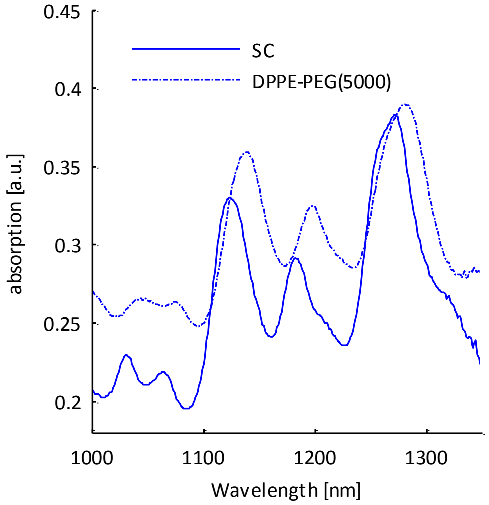

Supplementary Figure 9. SWCNT surfactant coating exchange. Absorption spectra of the initial sodium cholate (SC) suspended SWCNT and of DPPE-PEG(5000) suspended SWCNT following the removal of the SC by dialysis.

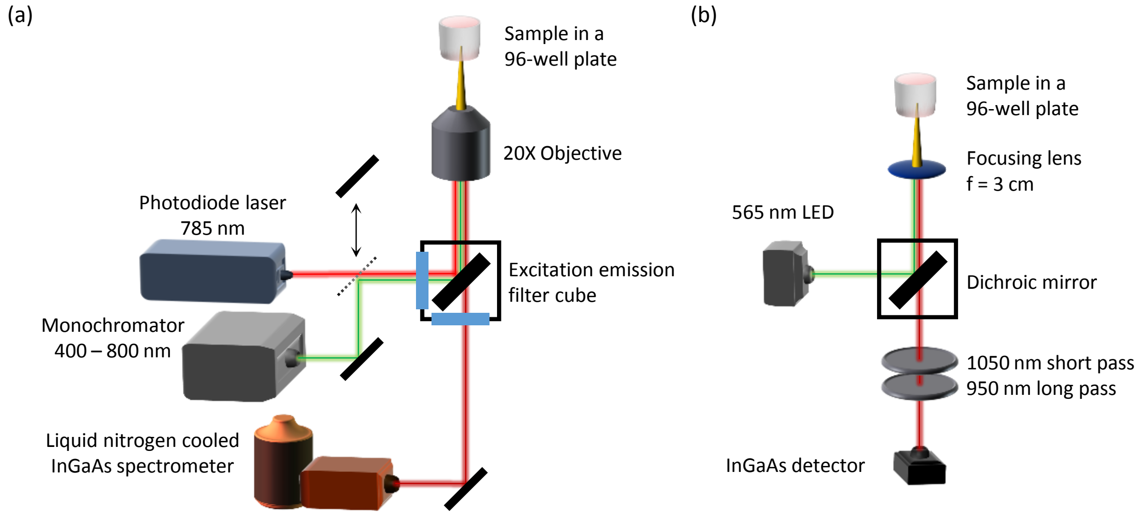

Supplementary Figure 10. Experimental microscopy systems. Optical setup of the nIR fluorescent microscopes for (a) high throughput screening and (b) immobilized SWCNT sensors detection.

Supplementary Table 1. Fit parameters for the solvatochromism shift model

Wrapping	Slope	R^2	ε_{eff}	α
**$(GT)_{15}$**	0.06600	0.972	16.316	0.854
**$(GU)_{15}$**	0.06740	0.974	18.958	0.822
**$(AT)_{30}$**	0.06481	0.942	14.581	0.874
**$(GC)_{30}$**	0.06735	0.974	18.842	0.824
**$(AT)_{15}$**	0.06922	0.963	23.933	0.763
**$(AAAT)_{7}$**	0.06958	0.971	25.258	0.747
**$(ATTT)_{7}$**	0.06782	0.976	19.918	0.811
**$(GGGT)_{7}$**	0.06846	0.978	21.576	0.791
**$(GTTT)_{7}$**	0.07096	0.966	31.882	0.668
**DPPE-PEG(5000)**	0.06346	0.983	12.999	0.855
**DMPE-PEG(5000)**	0.07091	0.964	31.564	0.657
**DSPE-PEG(5000)**	0.06332	0.982	12.859	0.875
**DSPE-PEG(2000)**	0.07175	0.990	37.417	0.589
**DSPE-PEG(2000)-Cyanur**	0.06525	0.987	15.188	0.848
**DSPE-PEG(2000)-CA**	0.07028	0.992	28.211	0.696
**DSPE-PEG(2000)-Maleimide**	0.06897	0.991	23.123	0.755
**DSPE-PEG(2000)-PDP**	0.06948	0.989	24.883	0.735
**DSPE-PEG(2000)-Amine**	0.06664	0.983	17.440	0.821
**DSPE-PEG(2000)-Biotin**	0.06716	0.988	18.459	0.810
**DSPE-PEG(350)**	0.07304	0.983	52.292	0.416

The slope, R-squared, effective dielectric constant (ε_{eff}), and the relative surface coverage (α).

Supplementary Note 1. Background on fibrinogen

Fibrinogen is an important biomarker, and its plasma concentration has clinical value. It is an acute phase reactant, a coagulation factor, and the principal element of blood clots. The fibrinogen protein is synthesized in the liver and is one of the most abundant proteins in plasma with normal concentrations[1] of 1.75-4.3 g L^{-1}. Increased fibrinogen levels can indicate inflammatory state, or pregnancy, whereas decreased levels can result from hepatic synthesis dysfunction, thrombosis, genetic diseases such as afibrinogenemia, hypofibrinogenemia, and dysfibrinogenemia, and disseminated intravascular coagulation[1]. The latter is associated with severe clinical conditions such as sepsis, trauma, cancer, obstetrical complications, vascular disorder, presence of toxins, and immunologic disorder[2].

The coagulation cascade associated with the wound healing response can be initiated by the activation of either one of two possible pathways, extrinsic and intrinsic, which converge to a common pathway, in which prothrombin is converted into thrombin which hydrolyzes fibrinogen, forming insoluble fibrin monomers that polymerize to form a thrombus[3]. The extrinsic pathway is initiated by the release of tissue factors through a broken blood vessel wall, whereas the intrinsic pathway is initiated by contact with negatively charged surfaces within a damaged vessel, such as exposed subendothelial tissue or collagen[4]. Direct methods for determining fibrinogen concentration in blood are based on immunological assays, such as enzyme-linked immunosorbent assays (ELISA), radial immunodiffusion (Mancini method), and gel electrophoresis followed by immunoblotting[5]. Supplementary functional assays for estimating the amount of active clottable fibrinogen such as thrombin time (TT), prothrombin time (PT), and partial thromboplastin time (PTT), involve the addition of an activator of one of the coagulation pathways to plasma samples, followed by a measurement of the clotting time[1,5]. Thrombin time tests the activation of fibrinogen to fibrin by adding high concentration of thrombin, prothrombin time screens the extrinsic pathway by adding tissue factors (thromboplastin) that are released upon vessel wall damage, and partial thromboplastin time screens the intrinsic pathway by adding ground glass to mimic the charged surface contact. Normal times are 20 – 30 sec for TT, 11-14 sec for PT, and 22-35 sec for PTT[6].

The fibrinogen protein is constructed by three pairs of mostly alpha-helix polypeptide chains (Aα, Bβ, and γ) linked by disulfide bonds. Its structure appears as three-nodules, one in the middle (E-domain), and two at each of the ends (D-domains), which are connected by helical chains[7,8]. The total length of the fibrinogen molecule ranges between 45 nm – 50 nm, the diameter of the middle and outer nodules are 5 nm and 6.5 nm, respectively, and the width of the interconnection helical coil is of the order of 1.5 nm[7]. The distribution of the possible angles between the two helical chains originating at the middle nodule was found to be bimodal with peaks around 157 and 106 degrees[9], granting the fibrinogen an elongated structure with high aspect ratio.

Supplementary Note 2. Protein panel

Our protein panel includes the following proteins: albumin[10], IgG[11], fibrinogen[12], α1-antitrypsin[13], Transferrin[14], Haptoglobin[15], α2-macroglobolin[16], IgA[17], IgM[18], α2-acid-glycoprotein[9], apopoliprotein A-I[19], insulin[20], human chorionic gonadotropin (hCG)[21], and C-reactive protein (CRP)[22]. The references indicate the corresponding protein data bank (PDB) files used for the visualization of the hydrophobicity maps.

Supplementary References

1 Nicoll, D., McPhee, S. J., Pignone, M., Chou, T. M. & Detmer, W. M. *Pocket Guide to Diagnostic Tests*. (Lange Medical Books/McGraw-Hill, 2000).

2 Levi, M. & ten Cate, H. Disseminated Intravascular Coagulation. *New England Journal of Medicine* **341**, 586-592, doi:doi:10.1056/NEJM199908193410807 (1999).

3 Mosesson, M. W. The roles of fibrinogen and fibrin in hemostasis and thrombosis. *Semin Hematol* **29**, 177-188 (1992).

4 Halkier, T. *Mechanisms in Blood Coagulation, Fibrinolysis and the Complement System*. (Cambridge University Press, 1991).

5 Mackie, I. J. *et al.* Guidelines on fibrinogen assays. *British Journal of Haematology* **121**, 396-404, doi:10.1046/j.1365-2141.2003.04256.x (2003).

6 Verhovsek, M., Moffat, K. A. & Hayward, C. P. Laboratory testing for fibrinogen abnormalities. *American journal of hematology* **83**, 928-931 (2008).

7 Yermolenko, I. S., Lishko, V. K., Ugarova, T. P. & Magonov, S. N. High-Resolution Visualization of Fibrinogen Molecules and Fibrin Fibers with Atomic Force Microscopy. *Biomacromolecules* **12**, 370-379, doi:10.1021/bm101122g (2010).

8 Scheraga, H. A. The thrombin–fibrinogen interaction. *Biophysical Chemistry* **112**, 117-130 (2004).

9 Schönfeld, D. L., Ravelli, R. B. G., Mueller, U. & Skerra, A. The 1.8-Å Crystal Structure of α1-Acid Glycoprotein (Orosomucoid) Solved by UV RIP Reveals the Broad Drug-Binding Activity of This Human Plasma Lipocalin. *Journal of Molecular Biology* **384**, 393-405 (2008).

10 Sugio, S., Kashima, A., Mochizuki, S., Noda, M. & Kobayashi, K. Crystal structure of human serum albumin at 2.5 Å resolution. *Protein Engineering* **12**, 439-446, doi:10.1093/protein/12.6.439 (1999).

11 Saphire, E. O. *et al.* Crystal Structure of a Neutralizing Human IgG Against HIV-1: A Template for Vaccine Design. *Science* **293**, 1155-1159, doi:10.1126/science.1061692 (2001).

12 Kollman, J. M., Pandi, L., Sawaya, M. R., Riley, M. & Doolittle, R. F. Crystal Structure of Human Fibrinogen. *Biochemistry* **48**, 3877-3886, doi:10.1021/bi802205g (2009).

13 Hyun Kyu, S., Kee Nyung, L., Ki-Sun, K., Myeong-Hee, Y. & Se Won, S. Crystal structure of an uncleaved α1-antitrypsin reveals the conformation of its inhibitory reactive loop. *FEBS Letters* **377**, 150-154 (1995).

14 Yang, N., Zhang, H., Wang, M., Hao, Q. & Sun, H. Iron and bismuth bound human serum transferrin reveals a partially-opened conformation in the N-lobe. *Sci. Rep.* **2** (2012).

15 Andersen, C. B. F. *et al.* Structure of the haptoglobin-haemoglobin complex. *Nature* **489**, 456-459 (2012).

16 Doan, N. & Gettins, P. G. W. Human α2-macroglobulin is composed of multiple domains, as predicted by homology with complement component C3. *Biochem J* **407**, 23-30, doi:10.1042/bj20070764 (2007).

17 Bonner, A., Furtado, P. B., Almogren, A., Kerr, M. A. & Perkins, S. J. Implications of the Near-Planar Solution Structure of Human Myeloma Dimeric IgA1 for Mucosal Immunity and IgA Nephropathy. *The Journal of Immunology* **180**, 1008-1018, doi:10.4049/jimmunol.180.2.1008 (2008).

18 Perkins, S. J., Nealis, A. S., Sutton, B. J. & Feinstein, A. Solution structure of human and mouse immunoglobulin M by synchrotron X-ray scattering and molecular graphics modelling: A possible mechanism for complement activation. *Journal of Molecular Biology* **221**, 1345-1366 (1991).

19 Ajees, A. A., Anantharamaiah, G. M., Mishra, V. K., Hussain, M. M. & Murthy, H. M. K. Crystal structure of human apolipoprotein A-I: Insights into its protective effect against cardiovascular diseases. *Proceedings of the National Academy of Sciences of the United States of America* **103**, 2126-2131, doi:10.1073/pnas.0506877103 (2006).

20 Fávero-Retto, M. P., Palmieri, L. C., Souza, T. A. C. B., Almeida, F. C. L. & Lima, L. M. T. R. Structural meta-analysis of regular human insulin in pharmaceutical formulations. *European Journal of Pharmaceutics and Biopharmaceutics* **85**, 1112-1121 (2013).

21 Lapthorn, A. J. *et al.* Crystal structure of human chorionic gonadotropin. *Nature* **369**, 455-461 (1994).

22 Shrive, A. K. *et al.* Three dimensional structure of human C-reactive protein. *Nature Structural & Molecular Biology* **3**, 346-354 (1996).
